# A Review of Destructive Methods for Assessing the Bulk Tensile Strength of Glass

**DOI:** 10.1007/s11340-026-01340-z

**Published:** 2026-07-14

**Authors:** W. Damen, T. Bristogianni, M. Overend, F. Oikonomopoulou

**Affiliations:** Department of Architectural Engineering and Technology, Delft University of Technology, Delft, The Netherlands

**Keywords:** Volumetric glass, Mechanical testing, Brittle solids, Tensile strength testing, Flaws, Destructive testing, Bulk flaws

## Abstract

**Background:**

There is an increasing demand for cast glass for load-bearing applications in the built environment. This material often contains bulk flaws; more so when for example waste cullet is used. A substantial obstacle to a wider application of such glass components is the lack of reliable methods for evaluating the effect of these bulk flaws on the mechanical performance of the material. Current standardized strength testing methods for glass focus on surface and edge characterization of thin, homogeneous elements.

**Objective:**

This paper identifies existing destructive methods with potential for measuring the tensile strength of glass and other brittle materials; and assesses to what level they give information about the bulk strength in the presence of bulk flaws.

**Methods:**

A literature review is conducted on existing destructive testing methods in use for brittle solids from various research domains and end applications. Existing methods are summarized, and their applicability for volumetric glass is critically assessed and discussed.

**Results:**

The selected methods are classified, based on stress state, stress distribution and stressed volume, accuracy, consistency and verifiability, complexity and precision required for the test specimens, and the availability of the required test equipment. Subsequently, the various advantages and limitations of the tests are summarized.

**Conclusion:**

Several recommendations are made. The direct uniaxial tensile test is recommended for testing bulk flaws under a uniaxial tensile stress state, while the ultrasonic tensile test is discussed as a promising alternative. For biaxial testing the sphere compression test is proposed. Further physical experiments are recommended to assess their reliability and practicality.

## Introduction

**Fig. 1:**
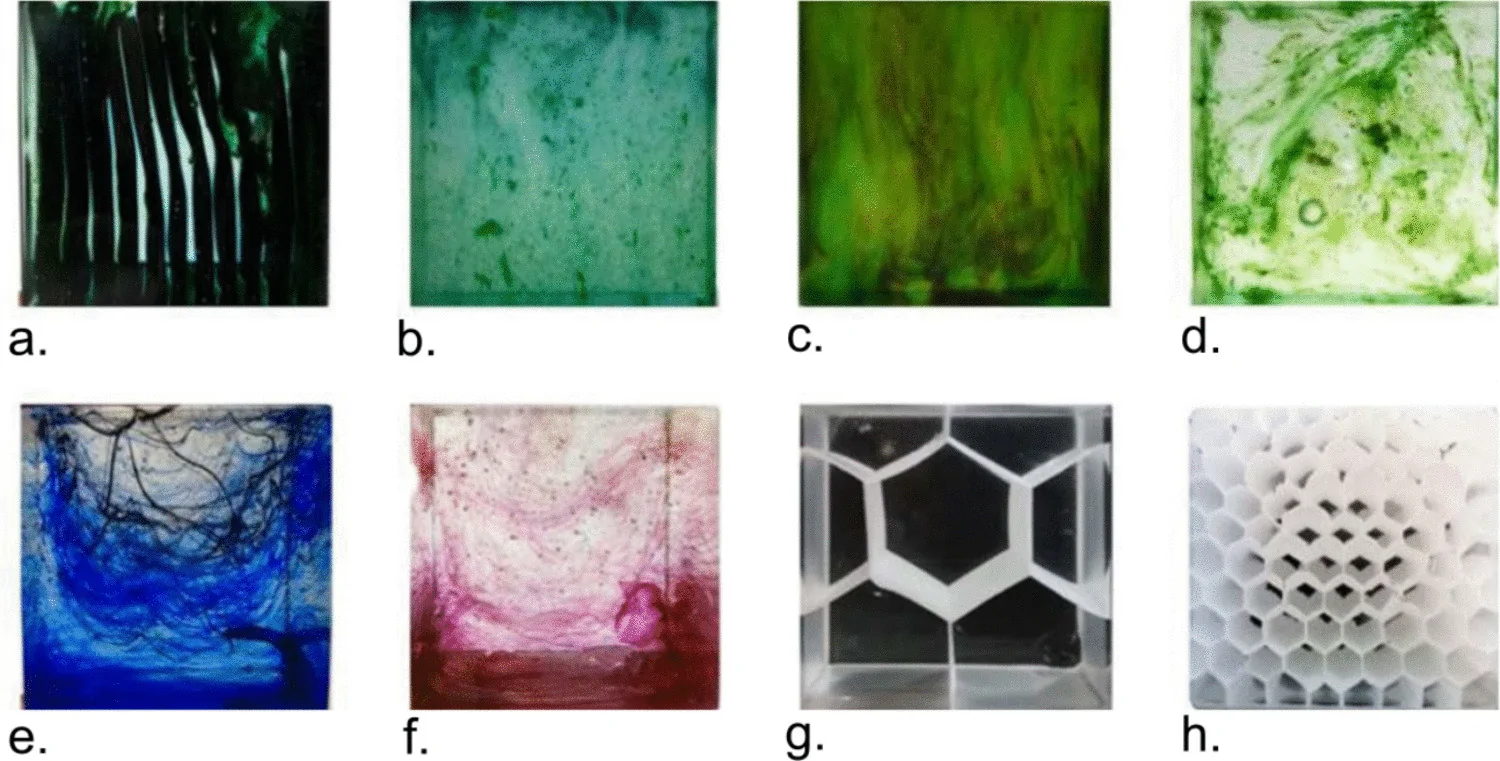
Kiln-cast glass specimens created using various composition of glass waste, each introducing different meso-level inhomogeneities and (bulk and surface) flaws. (**a**) float glass with enamel paint (**b**) car windshields (**c**) mixed container glass (**d**) oven doors (**e**, **f**) art crystalware (**g**, **h**) borosilicate rods

Cast glass is emerging as an alternative expression of architectural glass that enables the creation of three-dimensional, volumetric,^1^ robust glass components of large cross-sections that overcome some of the limitations of float glass by providing a wider variety of shapes and colors than is possible through other glass shaping methods (Fig. 1) [1, 2]. Furthermore, motivated by the transition a circular economy, recent research at TU Delft has demonstrated the potential of casting difficult-to-recycle glass waste streams into solid, volumetric components [1, 3–6]. The resulting recycled glass components accommodate a significantly higher contamination rate than thin-walled glass elements [1, 4], thereby offering a promising pathway for sustainable glass construction.

**Fig. 2:**
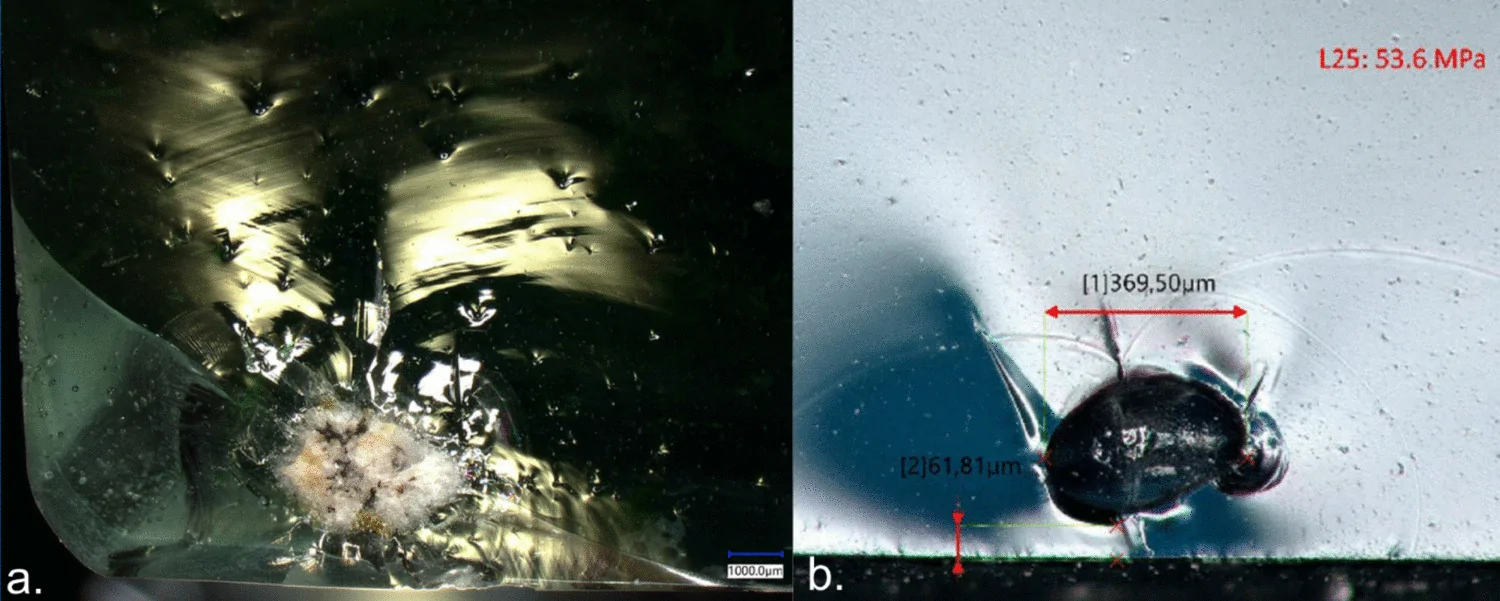
Examples of flaws in the material bulk of cast glass beams, resulting in fracture under four-point bending [4] (**a**) Crystalline inclusion in recast car window glass, (**b**) crystalline inclusion in recast rejected float line glass

A consequence of volumetric glass and its less automated production process [7], is the presence of bulk flaws, such as air bubbles, cords, and inclusions. These are various and considerable -both in size and population-, and can significantly influence structural performance. These flaws become even more pronounced when recycled glass, with higher levels of embedded contamination, is used as the raw material. Some examples of fracture of cast glass caused by flaws in the bulk are shown in Fig. 2. These samples were loaded in bending, resulting in peak tensile stresses -and consequently failure- originating from bulk flaws located in the vicinity the surface of the glass [4].

For glass, structural performance is governed by the tensile strength of the material, while compressive strength is around one order of magnitude higher than the tensile strength [8]. Consequently, even under compressive loading, failure will occur due to (indirect) tensile stresses [8, 9]. This is reflected in engineering design practice, where European and American design guidelines for glass in construction provide detailed recommendations on the tensile and flexural strength, but do not provide guidance on compressive strength [10, 11].

The mechanical testing of volumetric glass with significant bulk flaws has remained relatively underdeveloped. This is reflected by the fact that there is a complete lack of standards for establishing the strength of solid cast glass components [7]. Virtually all mechanical tests for structural glass are developed for float glass testing and are thus engineered for characterizing surface and edge flaws. In the words of glass artist and educator Liddleton “*we never test the strength of glass: all we test is the weakness of its surface*”. Bulk flaws, on the other hand, are generally considered negligible in float glass engineering [8]. Current testing methods therefore fail to assess how the mechanical properties are affected by various flaws in the bulk of the glass; nor do they show the effect of compositional variations of the glass itself.

There are multiple reasons for this focus on surface flaws. Due to the highly automated and controlled manufacturing process of float glass, surface flaws -usually encountered in the form of machining damage in post-processing (i.e. cutting, polishing), or damage obtained during use (i.e. scratches) [12]- are more common than bulk flaws. Mechanically, a surface flaw will have a higher stress intensity factor in comparison to a comparable bulk flaw [13], and glass elements subjected to bending will have the highest tensile stresses occurring at their surface. Finally, flaws located at the glass surface display subcritical crack growth (or ‘static fatigue’) due to humidity, which lowers their effective long-term strength [13, 14]. Bulk flaws, by contrast, are protected from these influences, and thus are not expected by the authors to display slow crack growth.

Previous research on the mechanical properties of cast glass included the testing of beams made of various glass waste streams through 4-point bending tests [1, 4]. These suggest that minor defects due to contamination, located in the bulk, have a limited, regulated effect in the Young’s Modulus and flexural strength of the components [1]. It was also found that recycled cast glass components can be further engineered into composite monomaterial forms, by featuring a core of lower-grade, higher-contamination glass and a surface layer of higher-purity glass [15, 16]. 4-point bending tests on such samples indicate mechanical properties almost comparable to prototypes made entirely of higher-quality glass [16, 17].

Despite these encouraging results, a major gap remains: the lack of reliable methods to evaluate how bulk flaws affect the structural integrity of cast glass components. This paper addresses this need by identifying promising tests that can be adapted for the testing of brittle solids containing significant bulk flaws, with a particular focus on cast glass. This supports the broader implementation of pure and recycled cast glass in structurally demanding architectural applications.

### Methodology and Scope

The aim of this paper is to identify existing testing methods that could be adapted for brittle solids containing significant bulk flaws, with a particular focus on cast glass. This is done through a review of existing destructive strength tests for glass and other brittle solids. This review examines a range of materials tested under varying conditions, including differences in specimen size, material quality, testing speed, and applied volume. As a result, meaningful quantitative comparisons are not possible, and the tests are compared qualitatively instead.

This review focusses on the fundamental behavior of bulk flaws in glass. Ideally, the chosen test should therefore produce fracture from bulk flaws over surface flaws, either by applying maximum tension to the material bulk, or otherwise limiting the occurrence of failure from the surface. As the aim is fundamental research rather than testing a specific end application with predetermined loads, boundary conditions and stress states, both uniaxial and multiaxial stress states will be considered. The primary use of the proposed test will be to characterize glass elements for the building industry, with intended element sizes ranging from standard masonry bricks to massive monolithic slabs of 1 m by 3 m. Additionally, it should be possible to follow the destructive test with fractography in order to identify the critical flaw at the fracture origin. This is particularly relevant for a material such as cast glass using recycled material, which can contain a wide range of different flaws caused by specific contaminations in the raw material. By knowing which flaws adversely affect mechanical performance, for example a choice can be made to allow or exclude certain contaminations during manufacturing. The review is limited to mode I failure (tensile crack opening), as this is the dominant source of fracture in highly brittle materials such as glass [13, 14]. 

Sect. "Background" provides some background on glass, flaws, and various criteria that determine the suitability of a testing method. This is followed by Sect. "Overview of testing methods" which describes the existing testing setups considered in this review and provides examples of recent research on these methods. Subsequently, in Sect. "Discussion", the testing setups are evaluated qualitatively, based on theoretical and practical criteria that were set out in Sect. "Background". The paper ends with recommendations for suitable tests in Sect. "Conclusion".

## Background

### Glass Properties

Glass is a brittle, amorphous solid. Its lack of a crystalline lattice structure prevents macroscopic plastic deformation. Therefore, glass behaves as a perfectly elastic material at a macro scale, fracturing when its tensile capacity is locally exceeded, generally at a stress concentration around a flaw. While the theoretical molecular tensile strength of glass is circa 32 GPa [18], in practice its engineering strength is several orders of magnitude lower [19]. This practical strength is governed by the size, orientation and shape of the most critical flaw present, in combination with the fracture toughness of the material [20]. Such flaws may fail even under compressive loading because of locally-induced or indirect tensile stresses, which results in an engineering compressive strength at least one order of magnitude greater than the engineering tensile strength.

Unlike ceramics, glass does not contain a grain structure. Due to the lack of an underlying microstructure, glass does not display rising R-curve behavior; i.e. as a crack propagates, there is no increasing resistance against crack growth [14].

### Flaws

Glass will contain various flaws and impurities that can affect the strength of the material. These can be categorized based on three regional categories: (i) surface flaws; (ii) edge flaws; (iii) bulk flaws [21]. In cast glass, all three regional categories of flaws are generally present. Each of these categories contains different types of flaws; a collection of flaws of the same typology is referred to as a ‘flaw population’.

**Fig. 3:**
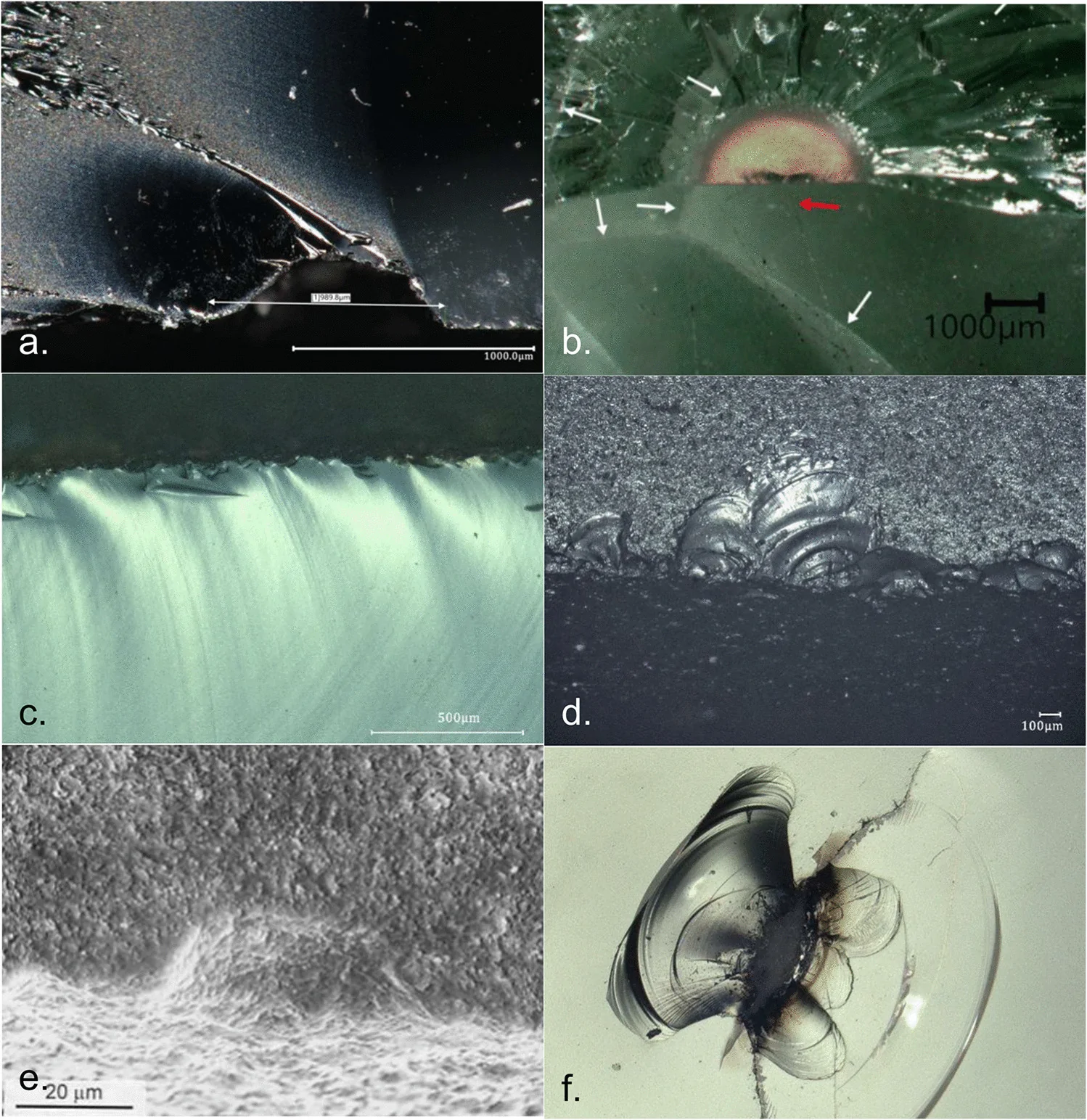
Various types of surface and edge flaws commonly encountered in glass, showing (**a**) an infold, (**b**) a surface grinding scratch, (**c**) subsurface cracks due to surface grinding, (**d**) edge chipping due to grinding, (**e**) Environmental exposure pit in silicon nitride (reproduced from [22] with permission of the author), (**f**) sharp object impact showing a pit, chipping and subsurface cracks

Surface and edge flaws are typically caused by the manufacturing process, or the subsequent handling or aging of the material. Based on their origins, the following classification can be made (Fig. 3):

- Glass forming damage such as surface voids (or infolds), caused by reactions between the glass and the casting mold or subsequent environmental exposure;
- Post-processing damage: including scratches, pits, cracks and chips, caused by cutting, grinding and polishing;
- Use damage: including pits and cracks caused by sharp or blunt object contact or impact, or environmental corrosion.

**Fig. 4:**
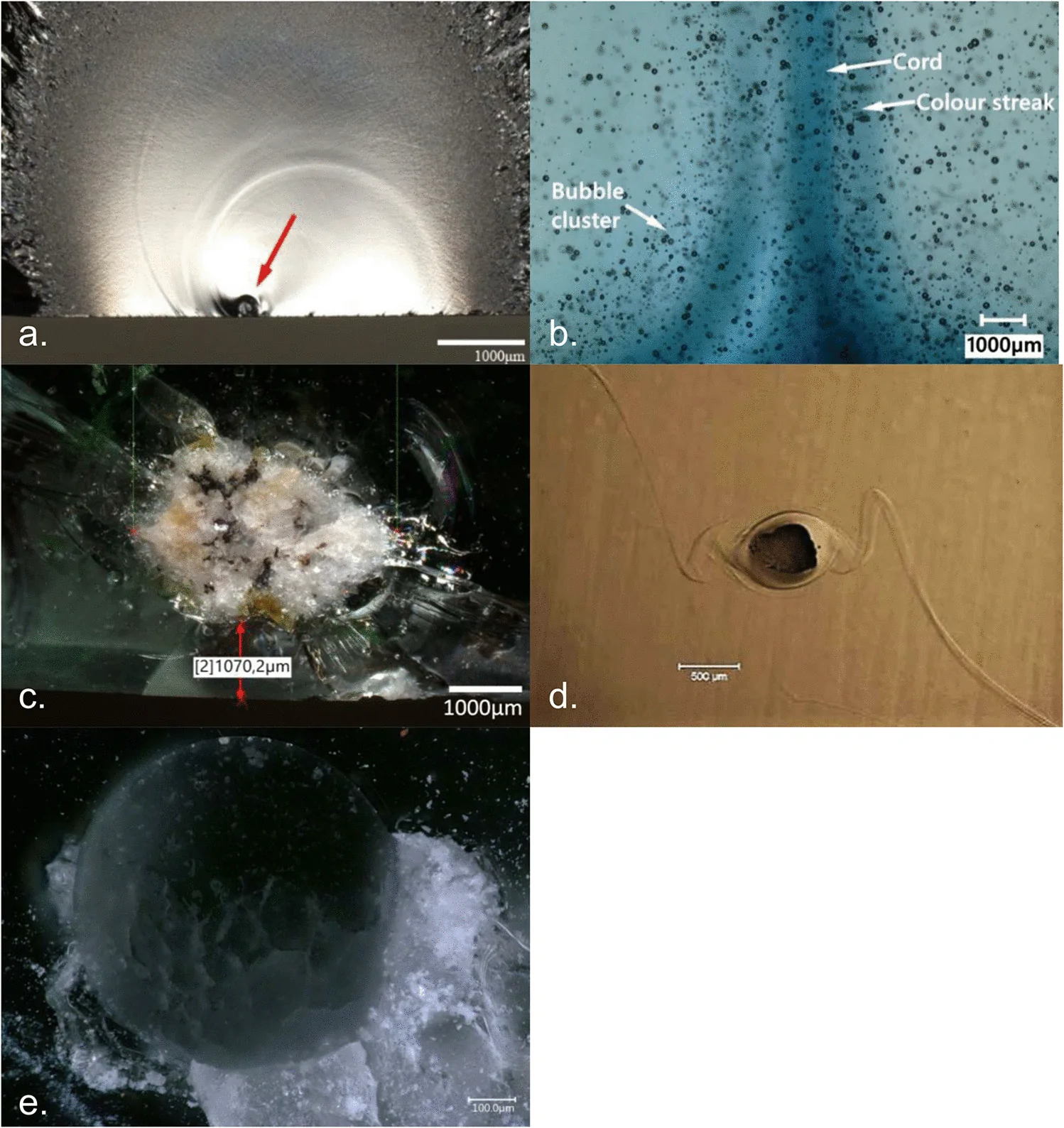
Various types of bulk flaws commonly encountered in glass, showing (**a**) surface-located bubble (**b**) cord and bubbles (**c**) crystalline inclusion (stone) (**d**) Stone with striae (reproduced from [22] with permission of the author) (**e**) bubble with crystallization (stone)

Bulk flaws are formed during the manufacturing process. Three main types are distinguished [7, 17, 23] (Fig. 4):

- Gaseous inhomogeneities, consisting of bubbles (or ‘seeds’) captured within the material;
- Crystalline inclusion (or ‘stones’), consisting of non-amorphous elements within the glass matrix;
- Glass inhomogeneities such as cords or striae, which are glassy inclusions of different composition from the glass matrix.^2^

It should be noted that bulk flaws can eventually be exposed at the surface of the material due to post-processing; for example, an inclusion can become exposed at the surface after cutting and grinding. When investigating glass fracture, it is therefore valuable to not only report the location of the fracture origin, but also the type of flaw in which led to fracture [22].

### Testing Considerations

Different testing methodologies vary in their precision and approach to evaluating material properties, with each method offering distinct levels of sensitivity and relevance to specific mechanical or structural behaviors. In specific, aspects such as stress state and distribution, or the amount of material subjected to a stress can vary significantly between setups, which affects the measurement results. This section goes into the different aspects that should be considered when selecting a testing methodology.

#### Inverse scale effect

**Fig. 5:**
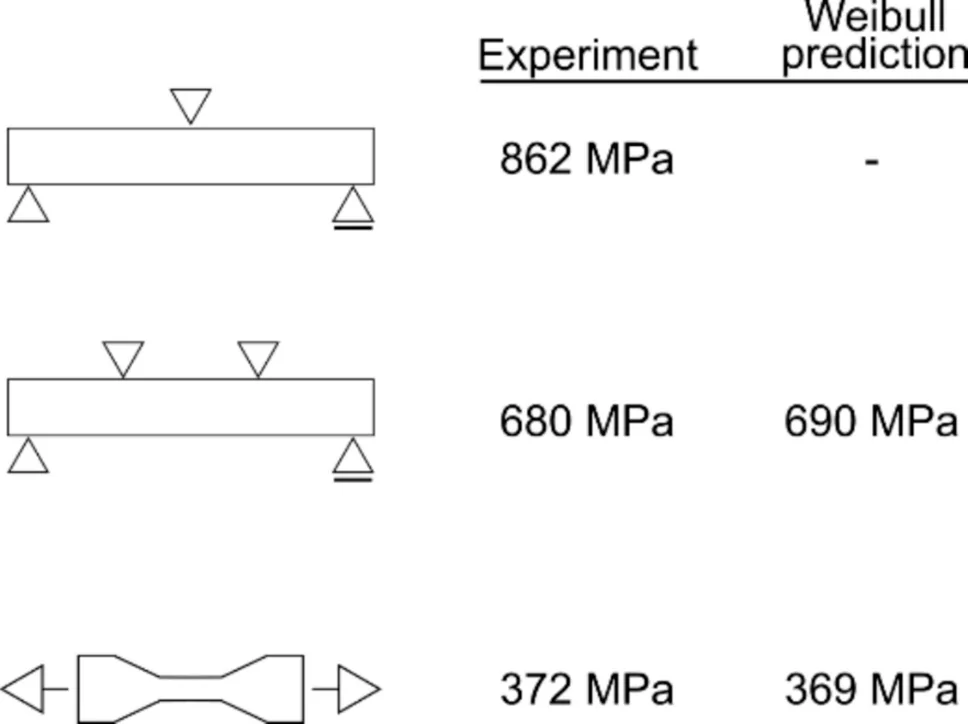
Example of strength measurements of one material using 3- and 4-point bending, and uniaxial tensile testing; illustrating how the measured values vary greatly due to the difference in loaded volume between each sample. In this case the variation is in line with predictions using Weibull strength scaling (adapted from [25] with permission from Wiley)

The strength of glass is governed by the presence of flaws. The greater the number of flaws that are subject to stress, the more likely it becomes that one of these will fail. Consequently, as the amount of stressed material increases, a lower strength will be found (Fig. 5).

In Weibull statistics, the relationship in strengths, between two components of different effective volumes V_1_ and V_2_ under uniform tensile stress is given by the following formula [24]:

1 $$\frac{{\sigma}_{1}}{{\sigma}_{2}}= {\left(\frac{{V}_{2}}{{V}_{1}}\right)}^{{~}^{1}\!\left/ \!{~}_{\beta }\right.}$$

With β being the Weibull shape parameter of a material. If strength is instead governed by surface flaws, volume is replaced by surface area. Consequently, following the square-cube law, the inverse scale effect generally more strongly affects bulk flaws over surface flaws; as the volume of a component changes faster than the surface area.

An implicit assumption for this relationship is that strength is governed by the same flaw type at both considered scales. This assumption becomes invalid if in fact a different flaw population becomes governing at the second scale. In such a case, the different flaw types/populations have different strength distributions associated with them, and will therefore scale in size differently [25]. Due to this, extrapolation of results to significantly different scales can be highly unreliable. When testing for a specific application, it is therefore best to test materials that closely resemble the final component in size and flaw distribution. If more generic data is required, ideally samples at several, preferably significantly different scales should be tested, to establish whether there are different flaw populations that govern strength at different scales. Additionally, these tests should be accompanied by fractography to determine the precise flaw type that is the source of fracture [22].

#### Stress distribution

When selecting a test for evaluating material properties in a specific area of a sample (in this case, the bulk strength), it is important take into account the applied stress distribution, as this determines the type and location of flaws that are triggered. The stress distribution within a test sample will vary between a uniform stress and a stress gradient, depending on the boundary conditions of the setup. Flexural tests generate a stress gradient with the highest tensile stress located at the surface of the material, which results in a bias for fracture from (sub)surface flaws on the zone subject to maximum stress; while a more critical flaw present e.g. on the top surface of a flexural specimen will not be triggered as the stress field in that location will be significantly less. If significant and diverse bulk flaws are present, as can be the case with recycled, volumetric, cast glass components, a uniform tensile stress distribution is more likely to trigger the most critical flaw, which could lead to fracture from the bulk.

In addition, the effect of the aforementioned scale effect needs to be considered, as the stress gradient influences the volume or surface area that is subjected to maximal tension. Weibull scaling can be used to account for this effect, by considering the effective area or volume loaded (Fig. 5).

#### Stress state

Different tests will generate different stress states in a material. These can be uniaxial or biaxial when considering sufficiently thin samples or material surfaces, or triaxial for a volumetric material. In practice, these stress states often result in different strength values for seemingly identical materials [26]. Generally, for brittle materials the multiaxial tensile strength will be lower compared to the uniaxial strength [27–29], though at least one case of biaxial strengthening in a glass–ceramic has been reported [30].

Generally, these difference in strength can be attributed to both statistical and mechanical effects [30]. Statistically, assuming a large number of flaws that have random orientations throughout the sample, a multiaxial stress state will cause a larger number of flaws being oriented most unfavorably for mode I failure, i.e. perpendicular to the stress direction. This increases the probability of one of these flaws being critically loaded, and subsequently failing [13]. From a fracture mechanics perspective, the failure criterion of certain flaws will change depending on the stress states, i.e. these flaws behave differently under uni- or multiaxial stresses, e.g. due to their orientation. The mechanical and statistical effects generally occur simultaneously, making it difficult to determine a precise relationship between uni- and multiaxial strengths.

To obtain an accurate design strength for a specific design application, the stress state used during material testing should resemble the expected stress state in the final object.

#### Accuracy and consistency

A testing setup should be accurate and consistent. It is critical that the measured values are representative for the property being measured. In the case of measuring tensile strength, this means that failure should be the result of mode I failure (crack opening). Additionally, if a gauge section is present, failure should consistently occur within this section. For this, it should be verifiable whether a test outcome is valid, for example through post-fracture analysis using fractography.

#### Fractography (post-fracture analysis)

Post-fracture analysis through fractography is a valuable tool for a better understanding of the numerical test results. It can often be used to locate the fracture origin, identify the critical flaw and follow the fracture path through the sample. Knowing the flaw type that led to failure is essential when investigating the precise role of different flaws, especially when multiple flaw populations are present within a sample. Additionally, when describing the results through Weibull statistics, each flaw population can be expected to have a different Weibull distributions. Therefore, to formulate a representative Weibull model of such a material, these flaw populations need to be distinguished and processed using censored statistics [22].

Furthermore, through fractography, slow crack growth can often be identified. Finally, the failure stress can be estimated by measuring the fracture mirror, if the fracture mirror constant for the specific material is known [22, 31].

#### Specimen complexity and dimensional accuracy

The dimensional accuracy and complexity of a sample that can be achieved is dependent on the production method used. Subsequently, when glass casting is used as a manufacturing method, certain fabrication limitations apply. Casting is performed through either kiln casting, generally using plaster molds, or hot-pouring using steel or graphite molds. Geometric deviations can be expected in both methods, primarily due to shrinkage of the glass during cooling. In cast glass bricks, a dimensional tolerance of ± 1.0 mm is expected, even when high-precision steel or graphite molds and low-expansion borosilicate glass is used [32]. Surface quality and dimensional accuracy of flat surfaces can be improved through grinding and polishing [33], while surfaces up to a certain curvature can be polished using specialized tools. A surface polish of up to 600 grit is recommended [4]. Glass elements can be milled using a glassblowing lathe, which has been used to make samples for tensile testing [34]. This is however a manual process, where the quality and consistency of the specimen is dependent on the skill of the operator.

## Overview of Testing Methods

An overview of existing research of selected destructive tensile testing methods is given. Examples of recent or notable works for the considered test methods are summarized in Table 1.

**Table 1:** Examples of recent or notable works for the considered test methods

Test method	Material tested	Source	Measurement goal	Specimen shape	Specimen size [mm]
Uniaxial tensile test	Boron Carbide (hot-pressed)	Swab et al. (2019)	uniaxial tensile strength	button-head dogbone	D = 3.0–6.35 (gauge section), l = 100–165
	**Glass (borosilicate)**	Alarcón et al. (1994)	uniaxial tensile strength	button-head dogbone	D = 4–8 (gauge section), l = 100
Three-point bending	Bone	Gebhardt et al. (2023)	E-modulus	non-uniform beam	h = ~ 2.26 w = ~ 9.52 l = 35.4
Four-point bending	**Glass (mixed waste glass)**	Bristogianni et al. (2021)	uniaxial flexural strength	rectangular beam	h = 20, w = 30, l = 300
	**Glass (soda-lime, reinforced)**	Cupać et al. (2021)	(post)fracture behavior, loading capacity	rectangular beam	h = 131, w = 25, l = 1400
Ring-on-ring	Alumina (99.5%)	Staudacher et al. (2021)	biaxial tensile strength	disk	D = 28.0 t = 1.0–1.7
	**Glass (aged soda-lime)**	Datsiou et al. (2025)	biaxial tensile strength	square panel	w = 160, l = 160, t = 4.0
Ball-on-ring	Alumina (99.8%)	Lupercio et al. (2021)	biaxial tensile strength	disk	D = 15.0 t = 1.5
	Silicon Nitride	Wiliams & Swank (1983)	biaxial tensile strength	disk	D = 82.6 t = 4.11
Ball-on-3 balls	Alumina (99.5%)	Staudacher et al. (2021)	biaxial tensile strength	disk	D = 28.0 t = 1.0—1.7
Pressure-on-ring	Alumina (99.5%)	Simpatico et al. (1999)	biaxial tensile strength	disk	D = 20.0–25.0, t = 0.635
	Aluminum Nitride	Simpatico et al. (1999)	biaxial tensile strength	square panel	w/l = 25.4, t = 0.66–0.81
Brazilian disk	Granite (Fangshan)	Li et al. (2020)	dynamic uniaxial tensile strength	disk	D = 50.0, t = 25.0
	Alumina (99.5%)	Chen et al. (2016)	dynamic uniaxial tensile strength	disk	D = 40.0 t = 18.0
	**Glass (soda-lime)**	Jiang et al. (2023)	dynamic uniaxial tensile strength	disk with flats	D = 9.0–18.0 t = 4.5
Theta specimen	Silicon (single crystal)	Gaither et al. (2011)	uniaxial tensile strength	round theta	D = 0.25, t = 0.025
	**Glass (soda-lime)**	Damen et al. (2025)	uniaxial tensile strength; test reliability	square theta	w = 150, l = 150, t = 10.0
Sphere compression	**Glass (soda-lime)**	Brajer et al. (2003)	biaxial tensile strength; failure mechanism	sphere	D = 1.0 to 8.0
	**Glass (soda-lime)**	Wang et al. (2020)	mode I-II-III crack growth	sphere	D = 80.0
Ultrasonic tensile test	Graphite	Pagnoncelli et al. (2024a)	uniaxial tension	dogbone	D = 5.0 (gauge section), l = 30.9
	Alumina (99.5%)	Pagnoncelli et al. (2024b)	uniaxial tension	rod	D = 10.0, l = 245
Spin test	GH 720 (nickel based alloy)	Xie et al. (2023)	RPM at failure	disk with rim	D = 155 t = 16.0
	Silicon Nitride	Wiliams & Swank (1983)	biaxial tensile strength	disk	D = 82.6 t = 4.11

### Uniaxial Tensile Test

A typical uniaxial tensile test considers an elongated sample clamped at both ends and pulled apart, generating a uniform tensile stress in the sample. The middle (gauge) section of the sample has a reduced cross-section, to ensure fracture occurs in this area. This test is widely used and acknowledged for a broad range of materials (e.g. for ceramics in ISO 15490 [35] and ASTM C1273 [36], and for graphite in ASTM C749 [37]). Besides strength, the uniaxial tensile test can be used to determine the elastic modulus and Poisson’s ratio of a material.

**Fig. 6:**
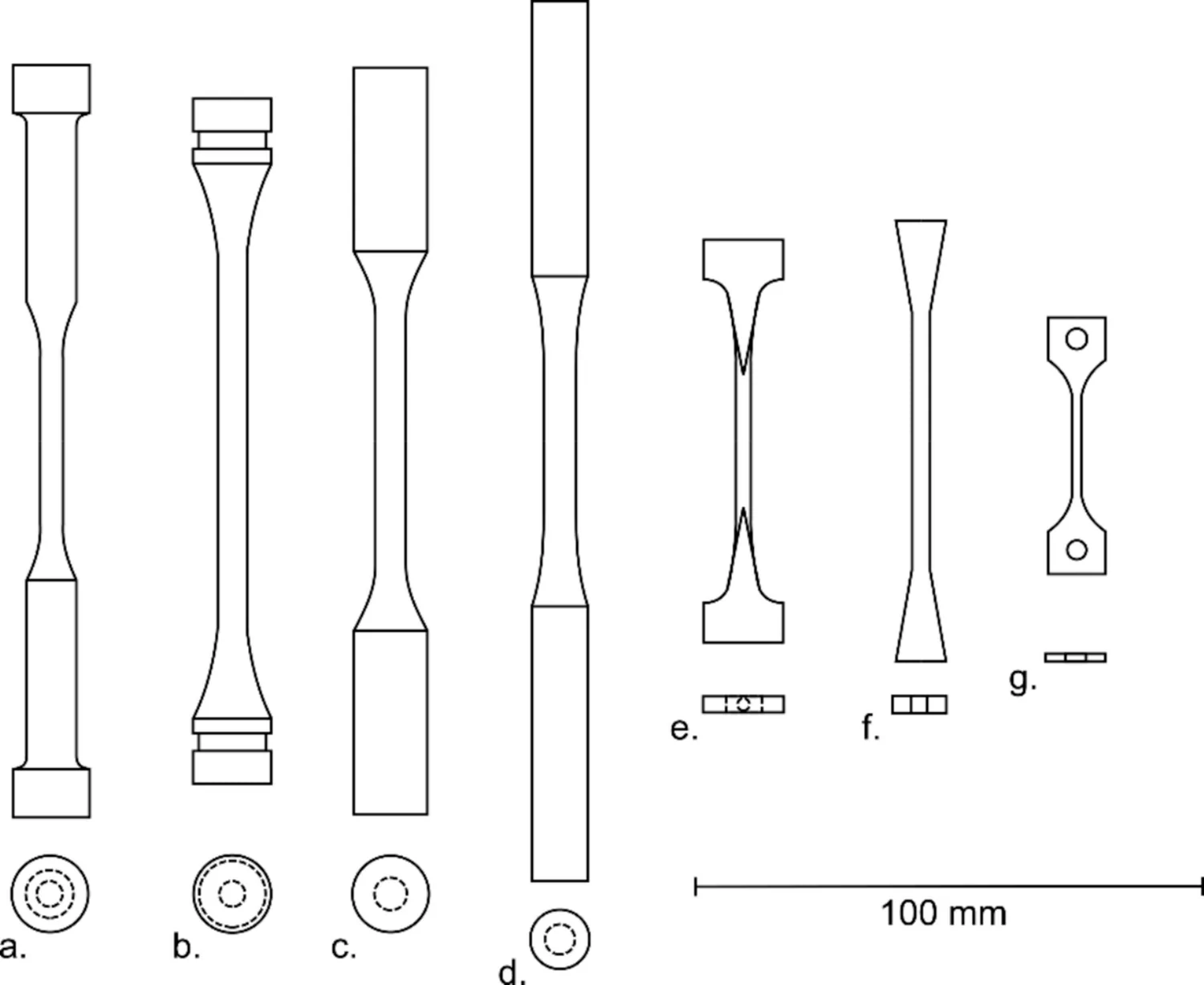
Examples of various uniaxial tensile samples for ceramics recognized by ASTM C 1273, showing (**a**, **b**) button-head samples, (**c**, **d**) straight-shank samples and (**e**–**f**) shoulder-loaded samples

**Fig. 7:**
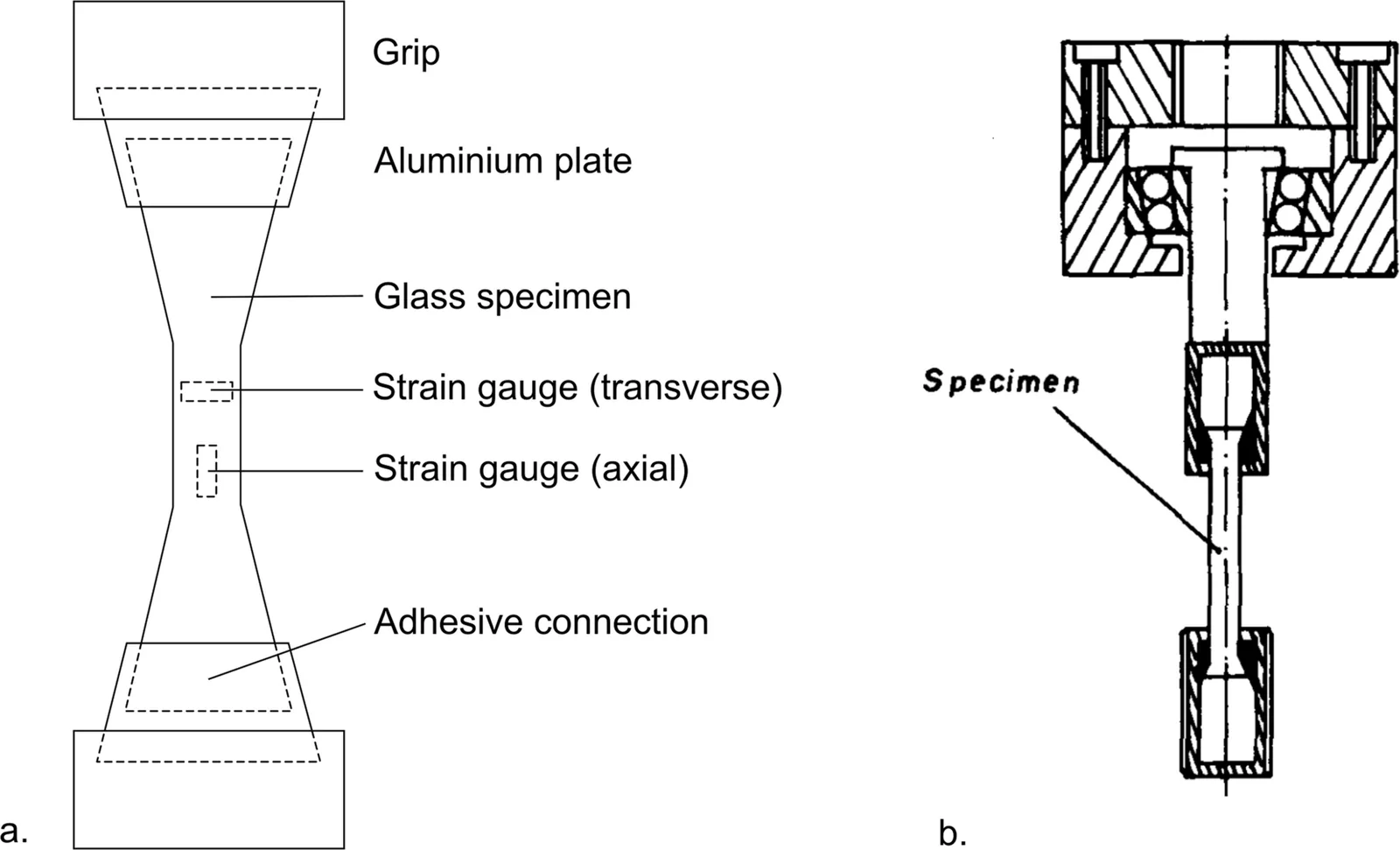
Uniaxial tensile tests on glass, showing (**a**) schematic illustration of a direct uniaxial tensile test as used in [40], using aluminium plates adhesively connected to 2 mm glass for loading, (**b**) dogbone sample fixed in metal end caps using epoxy, (reprinted from [34] with permission from Springer Nature)

A challenge of using this test for glass is the design of the interface between the sample and the testing machine. This can cause localized stress concentrations, which, in a brittle material, can lead to contact fracture, instead of fracture in the intended gauge section [38]. ASTM C1273 [36] describes various mechanical clamping mechanisms for ceramics [39], which might be adapted for glass testing (Fig. 6). An alternative is using glued connections, where the glass is glued to a metal support element that is subsequently attached to a testing machine. For glass, this method has been proposed for thin glass [40, 41], as shown in Fig. 7(a). [34] tested glass rods that were modified to a dogbone geometry using blowtorch lathe. Epoxy was used to fix these samples in metal supports that were subsequently attached to a UTM (Fig. 7(b)).

Another limitation of the uniaxial test is its high sensitivity to eccentricities that can cause unintended bending moments in the sample. This leads to deviations in measurements, and results in a bias towards surface fracture. This is especially noticeable in brittle materials that fail at a low strain, as this gives little opportunity for a stress gradient to redistribute to a uniform stress [42]. To reduce the effect of eccentricity, various self-aligning coupling systems have been developed; a comprehensive overview is given by [42]. However, due to friction effects, such systems cannot fully eliminate eccentricities, especially with low strength samples. Even in carefully designed and precisely machined samples, deviations due to misalignment of between 3—15% have been reported [42]. To detect unintended bending ASTM C1273 recommends using strain gauges [36], however this is relatively laborious. More recently, DIC has been used for detecting eccentricities [43]. The direction of eccentricity due to misalignment is difficult to control, and thus, to account for in the results. Deviations can be detected using multiple strain gauges per sample, however this is a relatively complicated and time-consuming measure when a large number of tests is performed. Due to these complications, in practice, flexural tests are often preferred by engineers [44].

### Three- and Four-Point Bending Tests

**Fig. 8:**
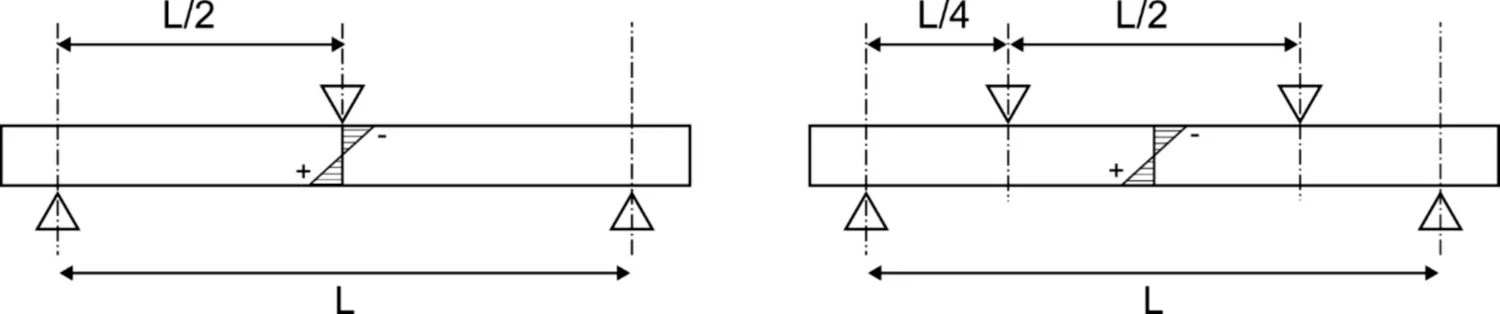
Schematic overview of three- and four-point bending tests as standardized by ASTM C1161; showing the stress gradient in the samples

Two types of uniaxial bending tests are widely used: the three-point and four-point bending tests. The four-point bending has been standardized for float glass in ISO 1288–3 [45] and ASTM C158 [46]. Both the three- and four-point bending tests have been standardized for ceramics: in the USA in ASTM C1161 [47] for rectangular beams and ASTM C1684 [48] for circular rods, and in Europe under ISO 14704 [49] (Fig. 8).

The three point bending test consists of a beam supported on two rollers, with a point load at the centre of its span. This results in a bending moment that is at its highest at the loading point and decreases linearly towards the supports. A limitation of this test is that the maximum tensile stress is limited to the part directly beneath the loading point, thus loading only a small part of the sample. Taking into account the inverse scale effect, this generally results in a relatively high measured material strength [50], as illustrated in the example at Fig. 5. The four-point bending test is similar in setup, loaded instead by two symmetrically placed bearings. This results in the sample being subjected to a uniform bending moment without shear in the section between the loading points (known as the load span). Generally four-point bending is preferable for deriving the engineering strength of a material over the three-point bending test, as more material is subjected to a peak tensile stress, resulting in a more representative measured strength due to the larger number of flaws that are loaded [44, 50]. Besides strength measurements, these tests can also be used to determine the elastic modulus of the material by measuring the displacement during loading, for example using sensors or digital image correlation (DIC).

Recent investigations into the mechanical strength of volumetric cast glass have predominantly employed four-point bending tests as the primary experimental method [1, 4, 5, 16, 51]. As expected by the imposed stress gradient, the majority of these tests resulted in fracture of the tested beams from flaws located at the surface; though several cases of fracture from flaws located in the bulk were found, in all cases due to crystalline inclusions just above the tensile surface (Fig. 2) [4, 16]. DIC was employed to validate measured deformations during testing [4], and, in the case of metal-reinforced cast glass beams, to monitor post-fracture behavior [51].

Due to the widespread use of three- and four-point bending tests, common causes of measurement errors have been extensively studied [25, 50, 52]. Both setups are sensitive to fracture at the sample edges due to machining flaws. This risk can be reduced by using arrised edges; however there is a risk of subsurface cracks remaining, or even new flaws being introduced by the lapping process [22]. Additionally, only a limited amount of material can be removed before the cross-sectional mechanical properties are noticeably altered. [52] contains an overview of the deviations due to lapping or filleting. The three-point bending setup is fully self-aligning (which allows for the testing of samples of nonuniform cross section [53]), while the four-point bending setup is more sensitive to geometric inaccuracies due to the additional contact point. Consequently, if the top and bottom surfaces of the sample are not parallel to each other, the two loading elements will not make simultaneous contact with the sample, resulting in an asymmetric loading configuration. This can mostly be prevented by using an articulated testing head for the testing machine. Fracture occurring predominantly under one of the loading points, or on the same half-side of the beams, can be a sign of this; though this can also be caused by local tensile stresses due to Hertzian contact (or wedging) stresses [52]. Additionally, if the beam sample is twisted along its axis, this results in deviations in stress from simple beam theory.

### Biaxial Flexure Tests

Flexure tests generate bending in a plate in two directions, resulting in a biaxial tensile stress at the surface of the plate. The loads are applied in the middle of a plate, as a result of which the outer edges are stressed relatively lightly, which reduces the sensitivity to edge fracture. Some circumferential stresses do however still occur, so fracture at the edges of the sample can still be observed [54, 55].

**Fig. 9:**
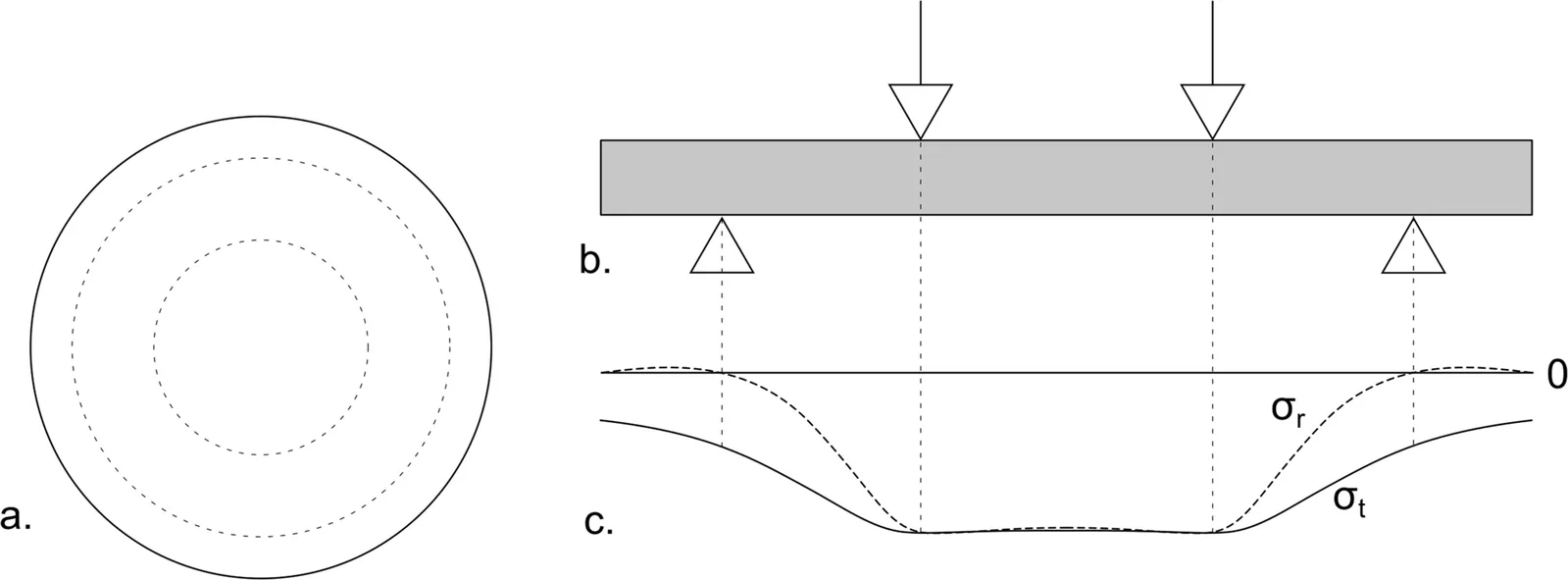
Schematic illustration of ring-on-ring test, showing (**a**) top view, (**b**) cross section, and (**c**) example of radial stress σr and tangential stress σt at the bottom surface

**Fig. 10:**
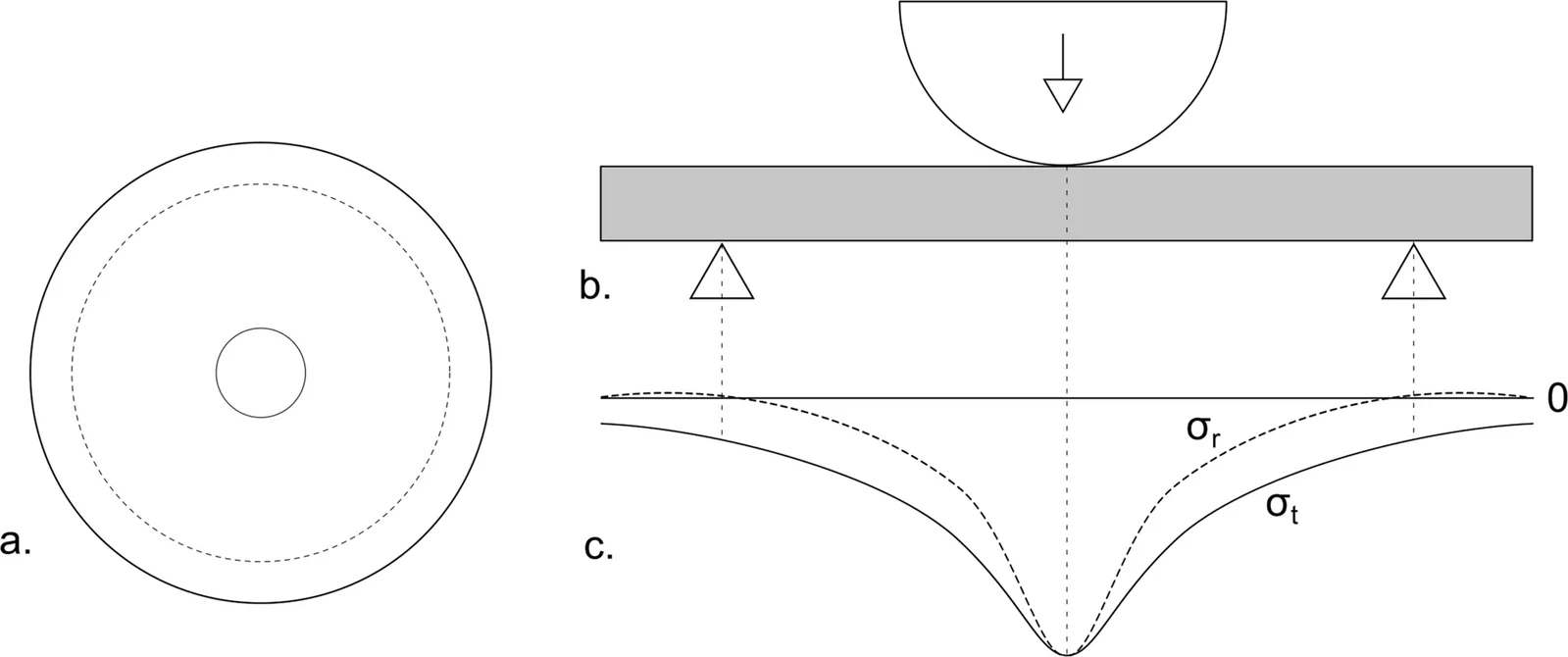
Schematic illustration of the ball-on-ring test, showing (**a**) top view, (**b**) cross section, and (**c**) example of radial stress σr and tangential stress σt at the bottom surface

**Fig. 11:**
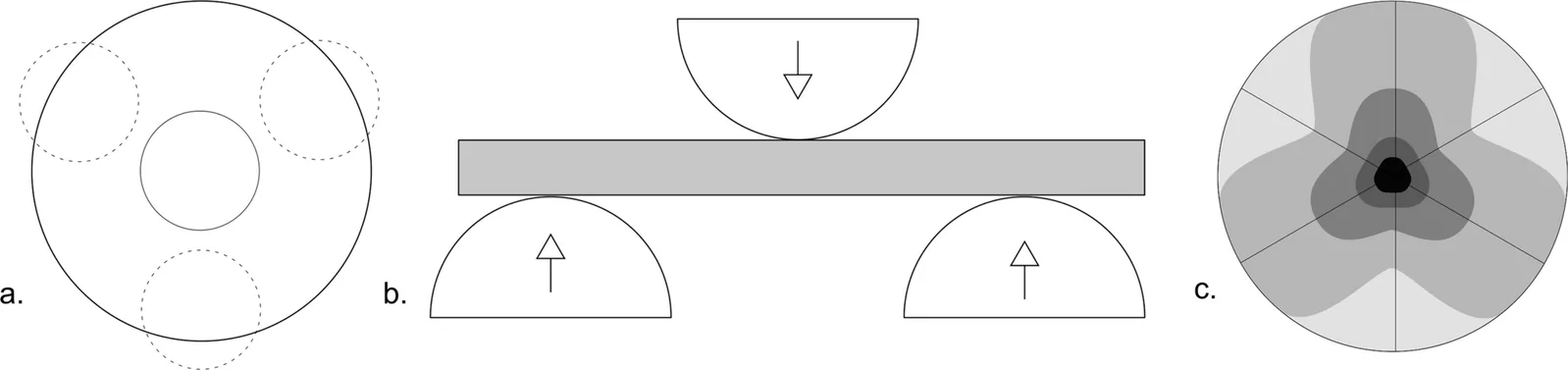
Schematic illustration of the ball-on-three ball test, showing (**a**) top view, (**b**) cross section, and (**c**) Example of maximum principle stress distribution at the bottom surface

**Fig. 12:**
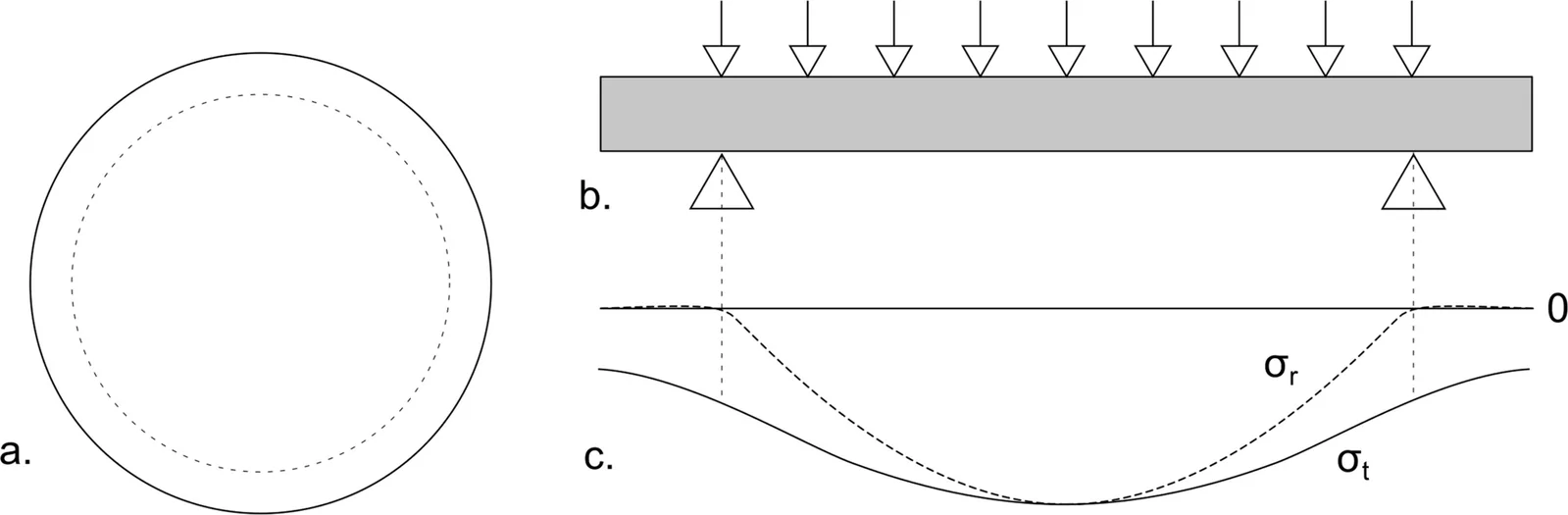
Schematic illustration of a pressure-on-ring test, showing (**a**) top view, (**b**) cross section, and (**c**) example of radial stress σr and tangential stress σt at the bottom surface

Various biaxial tests have been developed through the years. Some notable examples with the original publications:

- Ring-on-ring [56, 57] (Fig. 9);
- Ball-on-ring [58] (Fig. 10);
- Piston-on-three-balls [59];
- Ball-on-three-balls [60] (Fig. 11);
- Uniform pressure-on-ring [61] (Fig. 12).

A comparison between several commonly used biaxial bending setups have been made by [57, 58], while more recently an extensive review was presented in [54].

This category of tests places restrictions on the dimensions of the sample, which can limit the practicality if elements of a larger volume are to be tested. Specifically, the biaxial bending tests assume simple plate theory. To prevent membrane-like behavior, the deflection of the sample should be limited to approximately 0.5 times the sample thickness, which limits the minimum thickness of the sample. A large sample thickness, on the other hand, can lead to unintended wedging effects, resulting in unintended tensile stresses around the loading point. A recommended upper and lower thickness for a ring-on-ring setup is given in ASTM C1499 [62]; for soda-lime glass this corresponds to a thickness-to-support diameter ratio between 1:10 and 1:50.

#### Ring-on-ring test

The ring-on-ring test, also referred to as a Coaxial Double Ring (CDR) test, consist of a glass sample placed on a ring-shaped support, and loaded by a concentric, smaller ring. It can essentially be considered as a biaxial version of the four-point bending test. In this setup, the entire area within the inner ring is subjected to uniform, axis-symmetric biaxial tension. It is widely used, including for the testing of float glass, as the biaxial bending stress state resembles that of an out-of-plane loaded window pane, supported along its edges by a window frame. Relevant examples are presented at [63, 64]. It has been standardized for float glass in ISO 1288–2 [65] and 1288–5 [66], and for ceramics in the ASTM C1499 [62] and ISO 6474 [67].

A downside of this method is that the loading ring causes localized tensile stresses on the glass surface, directly opposite from the loading ring, caused by Hertzian contact stresses. This can potentially yield unreliable results due to premature failure under the loading ring. There is also a risk of friction at supports, though this can be reduced by replacing the continuous support ring by a ring of balls, or by using a low-friction foil or lubricant (as prescribed in the ASTM C1499). The ring-on-ring test requires a sample that is sufficiently flat to prevent asymmetrical loading by the rings and supports, though this requirement can be somewhat alleviated through the use of a soft rubber interlayer between the glass and the supporting and loading elements.

#### Ball-on-ring test

Ball-on-ring (BOR) test consists of a glass sample that is placed on a ring-shaped support, and loaded by a ball in the center of the ring. It can be considered a biaxial version of the three-point bending test. The sample is designed to be more self-aligning compared to the ring-on-ring test, which simplifies the testing process. This loading setup results in a symmetric stress distribution throughout the sample. However, only a very limited area is subjected to a peak stress. Additionally, the setup requires Hertzian deformation of the ball and sample to establish a contact surface; with the precise stress distribution under the loading ball being dependent on the applied load and the diameter of the loading ball [68–70] performed extensive BOR tests on alumina (99.8%) and zirconia (MgO and Y_2_O_2_ stabilized). The BOR tests for zirconia were in agreement with values from literature obtained from three point bending and piston/ball-on-three-balls tests. The results from alumina were on the low end of measurements from literature; the authors propose that this might be due to a difference in sample preparation and surface finish.

#### Piston on three balls/ball-on-three-balls test

Instead of a ring, these samples are supported on three balls and loaded with a piston or ball. This setup can accommodate samples that are warped or show other out-of-plane shape imperfections, as these will realign themselves on the supports. However, this does result in a non-axis symmetric stress distribution throughout the sample. The piston-loaded test was first introduced in [59], and was formerly standardized in ASTM F394. However, subsequent analyses by [57, 58] have shown that the stress state under the flat piston can be unreliable, with asymmetrical loading occurring when the piston and the sample are not fully perpendicular, and shifting contact surfaces as the sample deforms under the piston. The ball-on-three-ball test was described by [60] as an alternative that improves on this behavior. However, like the ball-on-ring test described earlier, this setup suffers from an uncertain Hertzian stress distribution under the loading ball [71] compared the performance of the ring-on-ring and the ball-on-three-balls setups on Alumina (99.5%). Weibull scaling was used to correct for the different stress distributions, resulting in comparable results from both setups for this material.

#### Uniform pressure-on-ring test

Also referred to as a ‘hydraulic burst’ test. This test is based around a specialized testing apparatus that uses pressured liquid behind a membrane to apply a uniformly distributed load on a ring-supported sample [61, 72]. It was designed for the testing of advanced ceramics. Due to the flexibility of the membrane, it can accommodate samples of uneven shape, and surface imperfections. In addition, the chance of localized fracture caused by loading mechanism is low. The resulting stress state in the material is axisymmetric, with a stress that is at its maximum at the center/bulk of the sample, decreasing towards the supporting ring. This test requires a specialized setup that is complex to set up and operate, in comparison to the other biaxial-flexure methods. It has therefore seen limited use and development since its introduction.

### Diametric Disk Compression (Brazilian Disk Test)

**Fig. 13:**
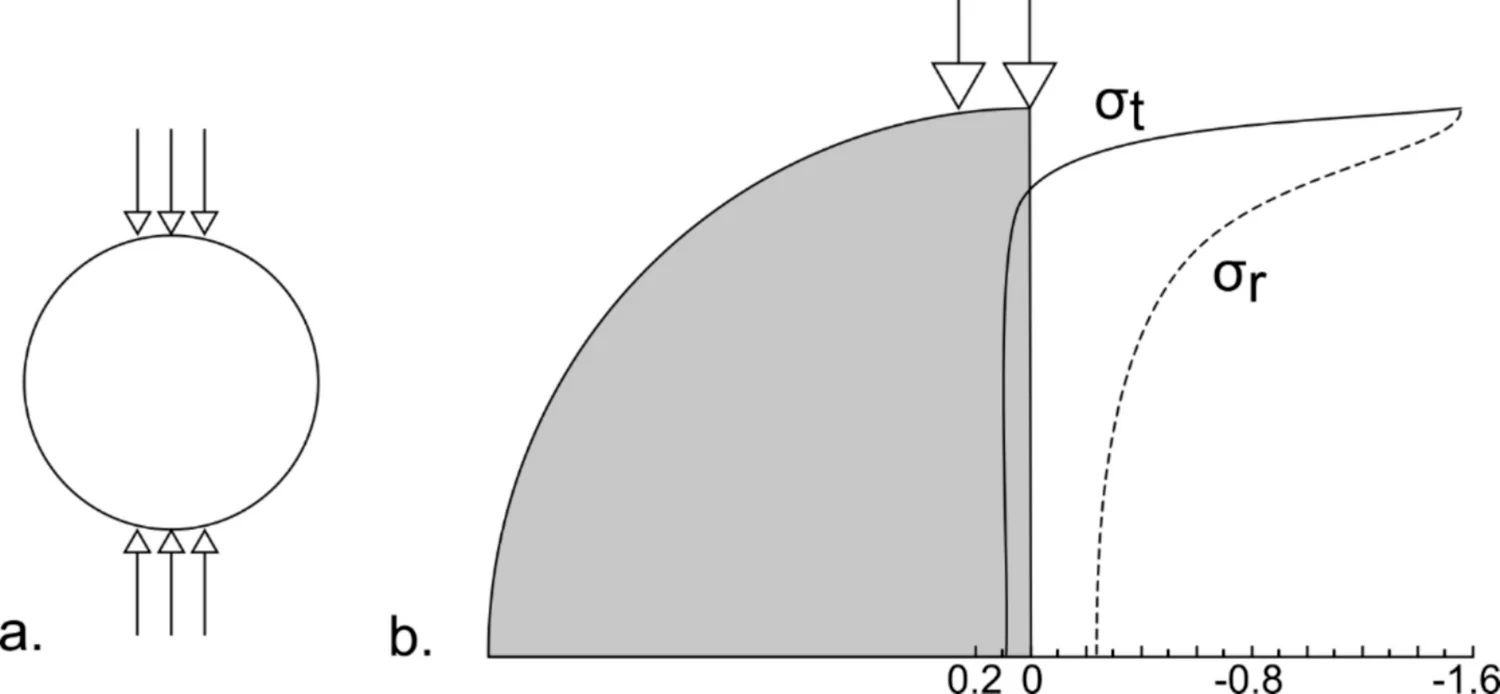
Schematic illustration of a diametric disk compression test, showing (**a**) schematic setup, (**b**) example of radial stress σr and tangential stress σt along the loading diameter of the disk (adapted from [76], with permission of Elsevier)

This indirect tensile test consists of a thick disk or short cylinder that is compressed diametrically between two parallel platens. The Poisson-induced sideways expansion of the material creates a zone of uniform, uniaxial tension in the center of the disk, perpendicular to the direction of the loading (Fig. 13). This methodology is standardized for the tensile testing of concrete in EN 12390–6 [73], graphite in ASTM D8289^3^ [74], and drilled rock core samples in ASTM D3967 [75].

The uniform tensile stress at the center of the disk will mean that both bulk, and surface flaws are loaded equally. Additionally, as the loaded surface is located at the center of the disk, the outer rounded edges do not require post-processing, as these lie outside the loaded zone. If the material bulk properties are to be tested, this reduces the chance of unintended surface fracture, and simplifies the preparation of the sample as no polishing of the rounded edges is required.

The tensile stress generated by the diametric compression is given by:

$${\sigma}_{x}= \frac{F}{\pi Rt}$$

where F is the applied load, R the disk radius, and t the disk thickness [50].

**Fig. 14:**
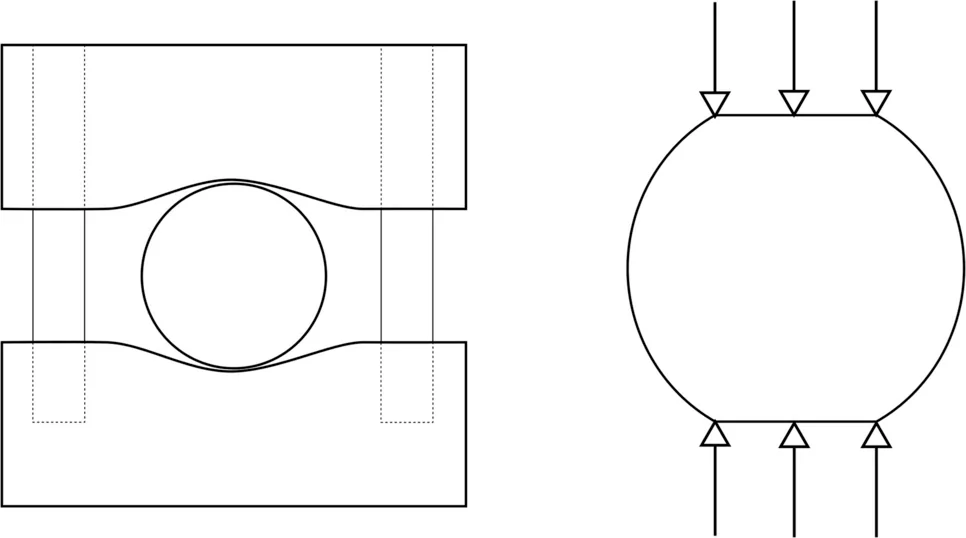
Schematic illustration of different methods of reducing the risk of fracture at the loading points, left: Semi-circular loading jaws as proposed by [76], right: Flattened disk geometry as proposed by [81]

The indirect tensile stress occurring is relatively low compared to the applied compressive force. Due to this, the elastic energy at the moment of fracture within the sample will generally be substantial, leading to explosive failure in ceramics and glasses. This makes it difficult to perform fractographic post-fracture analysis [76]. Related to this, different explanations of the precise fracture mechanism exist [77, 78] tested samples from sandstone, marble and granite, and found in all cases that cracks initiated at the surface directly beneath the loading points instead of the tensile zone in the center of the sample, and therefore questions the validity of this setup for measuring tensile strength, considering it more valuable as a comparison tool between different materials instead. The risk of fracture at the loading points can be reduced by introducing specialized curved loading jaws [74, 76], or a flattened disk geometry [79–81], which increase the contact surface between the sample and the testing machine (Fig. 14). EN 12390–6 [73] for concrete notes that different soft interlayers affect measured strength, and prescribes the use of hardboard strips, as this resulted in the lowest deviations in measurements during exploratory testing. Despite these measures, the often explosive failure of the samples makes it difficult to determine the precise origin of fracture, which in turn makes it challenging to determine whether a valid tensile fracture in the disks center occurred.

Jiang et al. [79] used a high-speed camera to record fracture of flattened soda-lime glass disk samples in both static and dynamic loading, finding a crack initiating at the centre of the sample, propagating to the loaded ends, concluding that this setup results in valid fracture. Similarly, [82] used DIC to record dynamic loading tests on irradiated granite disk samples, finding valid failure from the sample centre.

During testing, this setup also generates a compressive stress perpendicular to the tensile stress, equal to three times the tensile stress [50]. As a result, the stress state is biaxial, which can lead to different tensile strength values compared to a purely uniaxial tensile test [78].

### Theta (Θ) Specimen

**Fig. 15:**
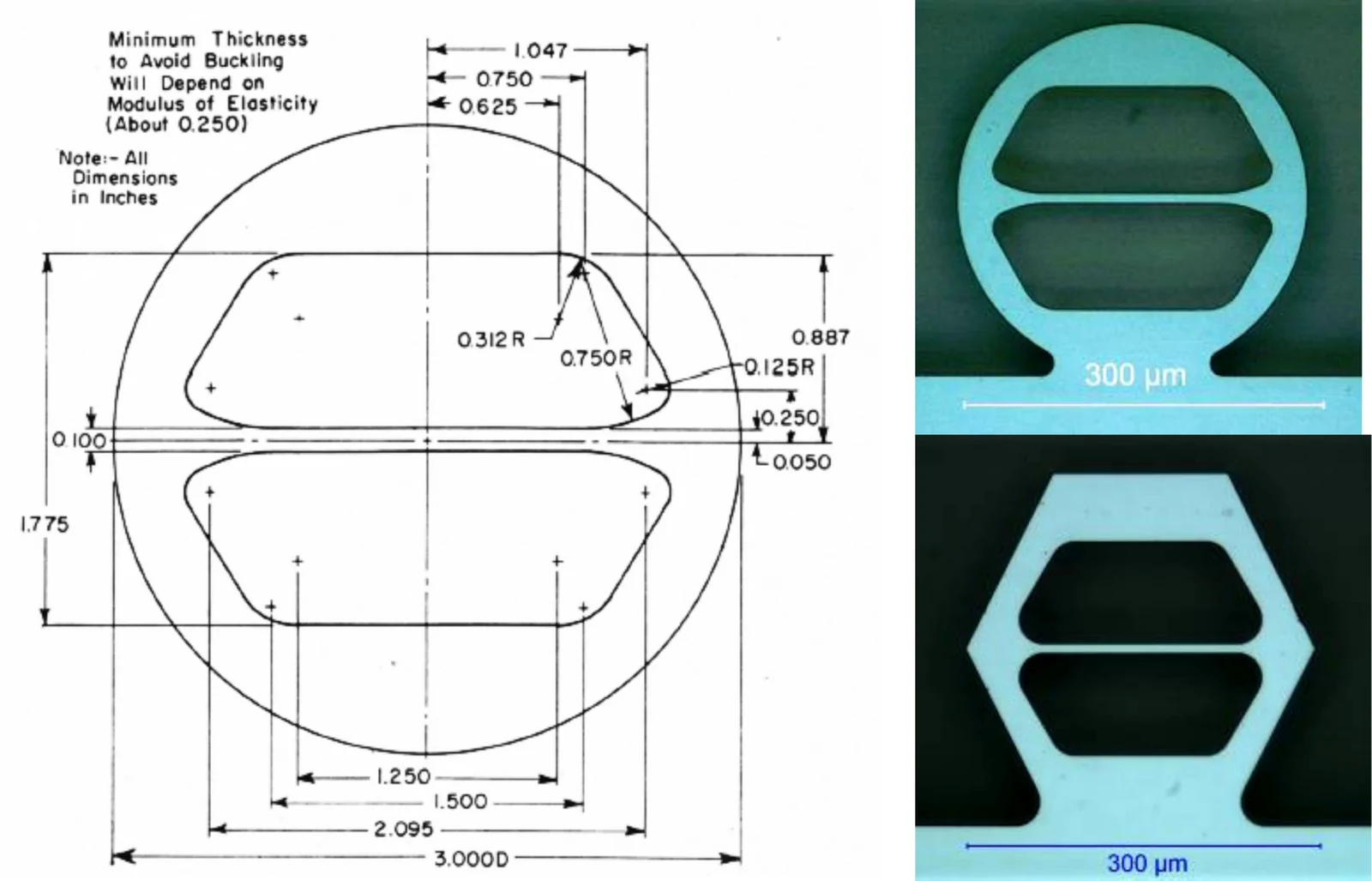
Theta sample: left: original geometry, (reprinted from [83] with permission of ASTM), right: Two versions of the Theta geometry for testing microelectronic elements (reprinted from [84] with permission of the author)

This sample was introduced by [83] as an alternative uniaxial tensile test for brittle materials, avoiding complex gripping that can lead to localized fracture at the supports. It consists of a disk with two added voids, shaped like the Greek letter Θ, so that a thin strip remains at the center of the sample (Fig. 15). When applying a diametric load on the disk perpendicularly to the strip, the sides of the sample are pushed outwards, generating a uniform uniaxial tensile stress within the central strip. It can be considered a hybrid design between a diametric disk and a uniaxial tensile test. Unlike a diametric disk test, the tested area is under pure tension without a perpendicular compressive force acting on it. The Theta specimen has been used for the testing of samples in micromillimeter scale for use in microelectronic systems, as shown in Fig. 15 [84, 85].

Recently, the Theta specimen was applied to glass [86]. A new square geometry was proposed to decrease stresses outside the central gauge section, while increasing the volume of material tested. This design was analyzed through FEA, and physically tested using samples cut from soda-lime samples using a waterjet cutter. Transient strains were visualized using polarized light, which revealed an unintended stress gradient in the gauge section of some samples. This effect was minimized by applying a soft neoprene interlayer between the glass and the testing machine, combined with more precise alignment of the samples. Measured strengths were relatively low for this material due to the rough surface finish of the waterjet-cut surfaces, which were difficult to post-process due to the sample geometry.

A drawback of this test is the relatively complex sample shape, requiring a significant amount of shaping and (post)processing. Additionally, only a small portion of the sample is tested, with much of the material in the sample essentially being underutilized. Mechanically, various bending moments occur outside of the tested strip. This leads to fracture outside of the tested strip, therefore rendering the test invalid. As the sample will break into multiple components it can be difficult to determine the origin of fracture [84], potentially requiring additional validation, for example through fractographic analysis [86] used fracture mirror measurements to estimate failure stresses at all fracture points, and observed that the lowest failure stresses consistently occur in the gauge section. They argue that this an indication of first fracture occurring here, with secondary high bending moments occurring outside of the gauge section as the load on the sample is rapidly redistributed after failure of the gauge section.

### Diametric Sphere Compression

This test is an extension of the diametric disk compression test, using a full sphere instead of a disk. The origins of this test lie in the testing of rock samples, where glass was used as a stand-in for rock materials [87–89]. Follow up research focused on glass spheres in 0.5—8 mm range, used as a fine grinding agent [90–92].

**Fig. 16:**
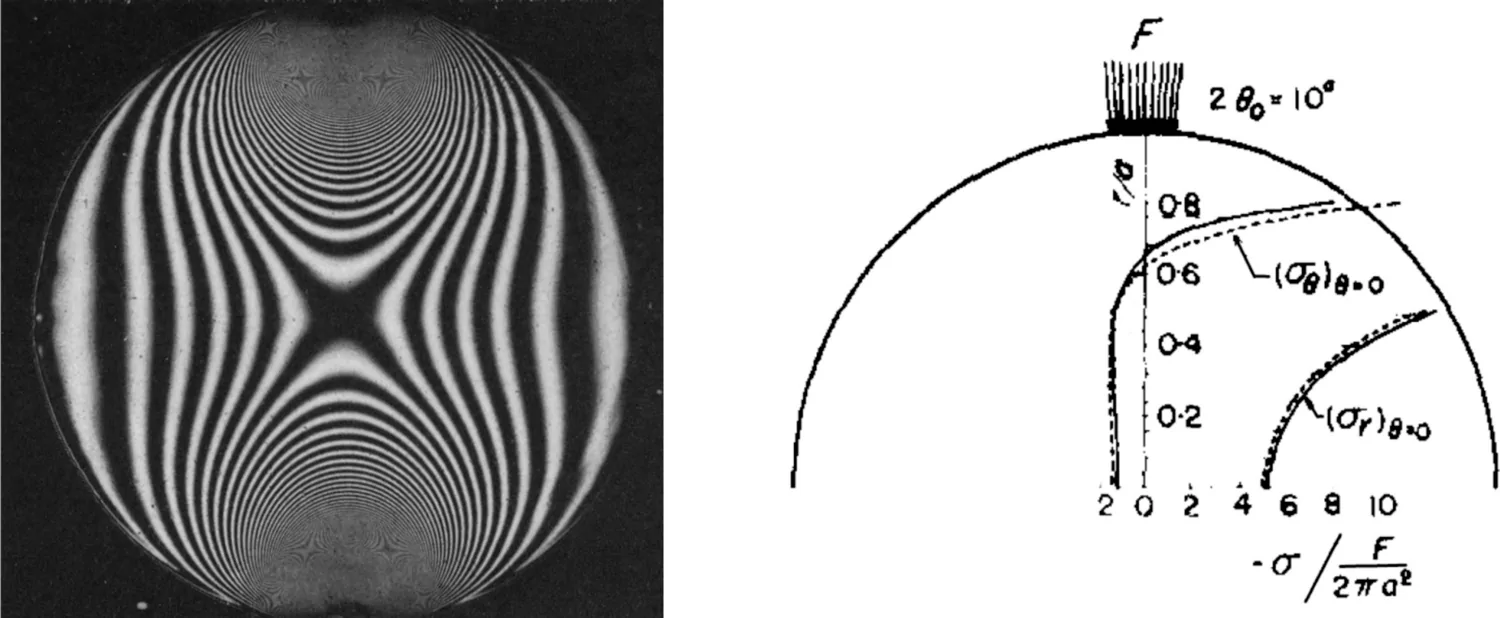
Diametric sphere compression samples (reprinted from [89] with permission of Elsevier). Left: photoelastic stress pattern in an epoxy-resin spherical sample, right: Distribution of principle stresses σθ and σr along the central axis, with σθ showing a near-uniform tensile stress at the center of the sample

Diametric compression of a sphere results in an equibiaxial tensile stress in the core of the sample, perpendicular to the applied load (compared to a uniaxial tensile stress when a disk is used). This test is unique in that the main tensile stresses lie fully within the bulk of the material, theoretically allowing for the testing of bulk properties without interference from the material surface (Fig. 16) [89].

**Fig. 17:**
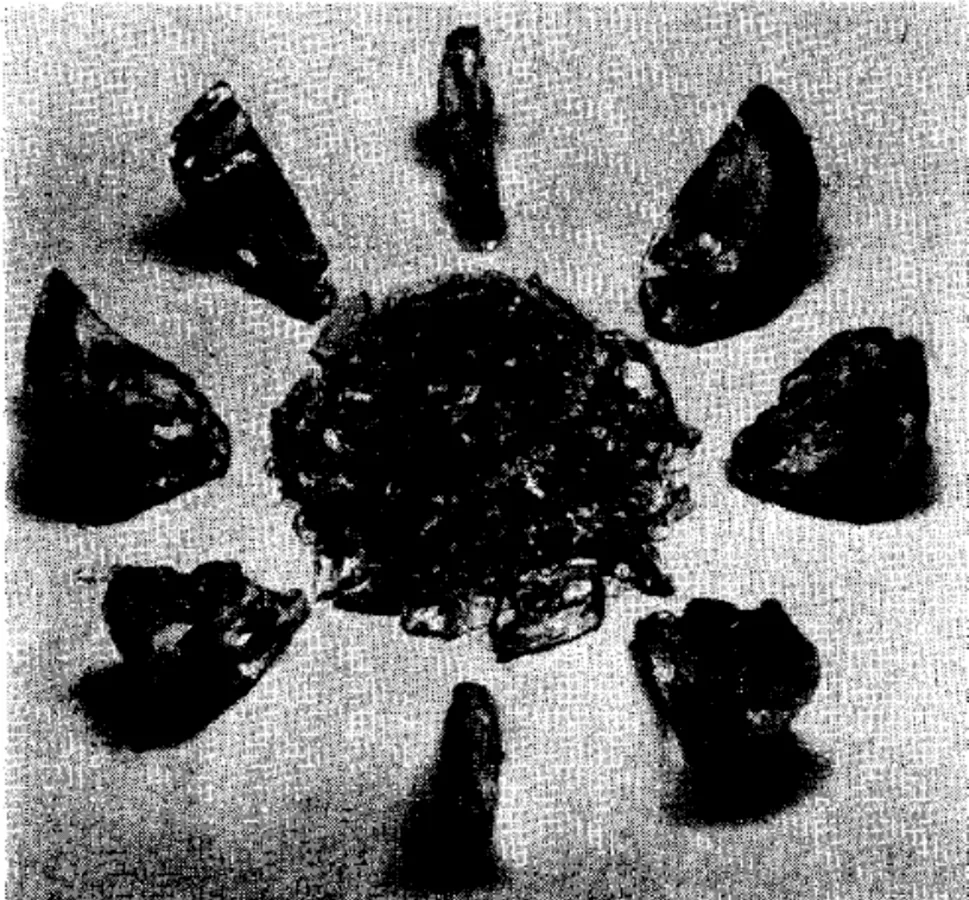
Fragments of a glass sphere recovered after fracture under diametric compressive loading, where gelatine was used to arrest the fragments after explosive failure. Crushing of the material between the diametric loading plates can be observed (reprinted from [88] with permission from AIP publishing)

However, in practice, it can be difficult to ascertain the precise fracture origin, due to the tendency of these spheres to fail in an explosive manner, which prevents the use of fractographic analysis. As of yet, there is no consensus on the dictating fracture origin in glass spheres. Several contradicting explanations have been proposed, arguing that fracture initiates either due to tensile stress in the material bulk [89, 90], shear failure at the supports [88, 92] (Fig. 17), or Hertzian contact cracking at the supports [93].

[94] used spheres to investigate mixed I-II-III fracture of glass. They tested both undamaged glass spheres (d = 80 mm), and spheres containing an internal penny-shaped crack (d = 15 mm) at a 60° angle, created through internal laser engraving. They observed that the undamaged sphere was fully pulverized on failure, with an unclear origin of fracture. The introduction of a penny-shaped flaw reduced the fracture load by 87%. Gradual extension of the internal crack towards the loading point could be observed during loading, showing that the internal flaw was successfully loaded in biaxial tension.

### Ultrasonic Tensile Testing

**Fig. 18:**
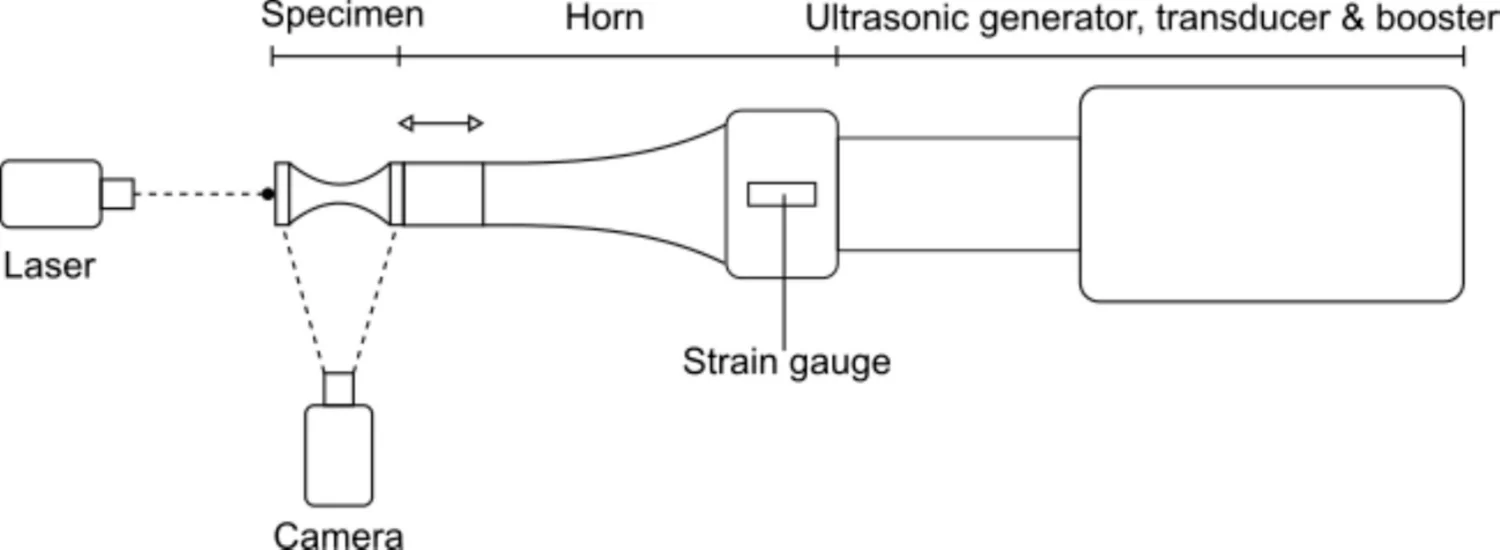
Schematic representation of the various components of the UST setup proposed in [96]

**Fig. 19:**
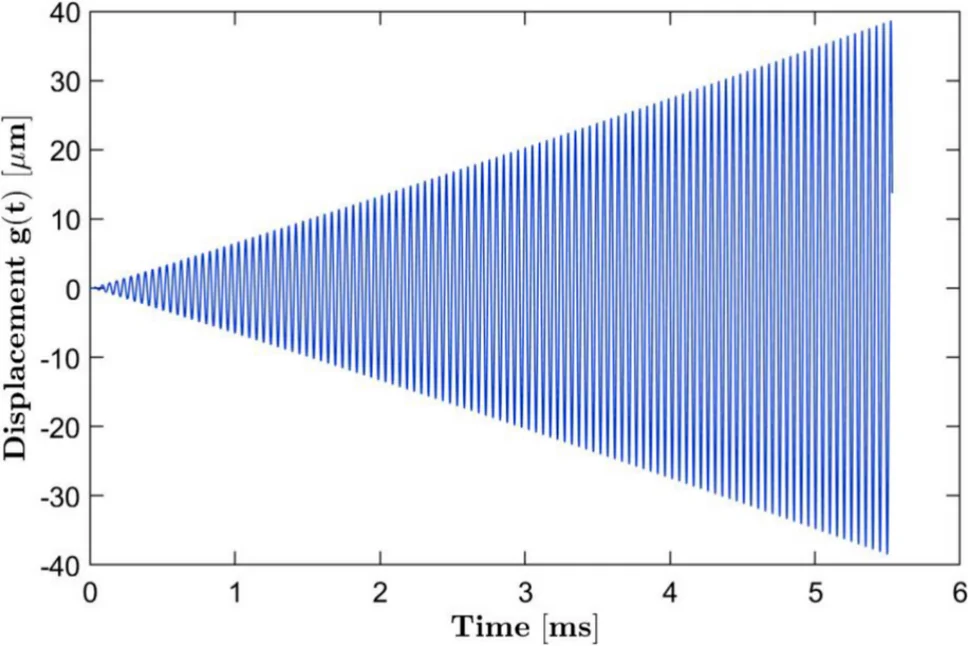
Time-displacement graph of the free specimen end during UST testing (reprinted from [97] with permission of the authors)

Ultrasonic fatigue testing machines (UFTMs) are widely used for investigating the Very High Cycle Fatigue response of materials, including brittle solids [95–97] adapted the UFTM for a quasi-static ultrasonic tensile test (UST) for brittle materials (Fig. 18). By rapidly ramping up the amplitude of the ultrasonic loading device (Fig. 19), hourglass-shaped graphite [96] and bar shaped alumina samples [97] were fractured in less than 100 loading cycles, which is fast enough to avoid fatigue damage. The cyclic loading results in alternating tension–compression stress, resulting in tensile failure. The mechanical properties obtained using this method were close to values found in the literature [96, 97]. An advantage of this method is the fact that very little direct loading is transferred from the testing device to the sample, as loads are generated by resonance of the sample itself. As a result, a relatively light adhesive connection between the sample and testing device can be used, minimizing challenges with gripping. The setup also has limited sensitivity to eccentricities. Additionally, this setup should allow for the test sample to be scaled up with few additional challenges, allowing for the testing of a relatively large material volume.

A drawback of the method is the relatively complex, multi-step numerical procedure required to extract the material properties from the measurements. The test is recorded using a combination of a laser measuring longitudinal deformation, strain gauges, and a high-speed camera to determine deformation using DIC (Fig. 19). These measurements are used to determine the displacement curves of the sample, and the number of cycles until the sample fractures. Different measurements are compared to minimize noise in the data. The measurements are then applied to a FEM model of the sample. Finally, the material properties of the model are optimized until they show satisfactory correspondence with the measured results.

It should be noted that this test causes localized crushing damage around the fracture surface, which makes it difficult to perform fractography. Instead x-ray CT scanning of the samples was performed before and after fracture to try to capture the fracture origin, and the role of different flaws within the material [97].

This testing setup demonstrates promising capabilities for the characterization of brittle materials, addressing the previously discussed open issues—particularly those related to gripping failures. Accordingly, the UST can also be applied to the testing of glass materials, as confirmed by ongoing joint activities with the authors of [97].

### Spin Testing

**Fig. 20:**
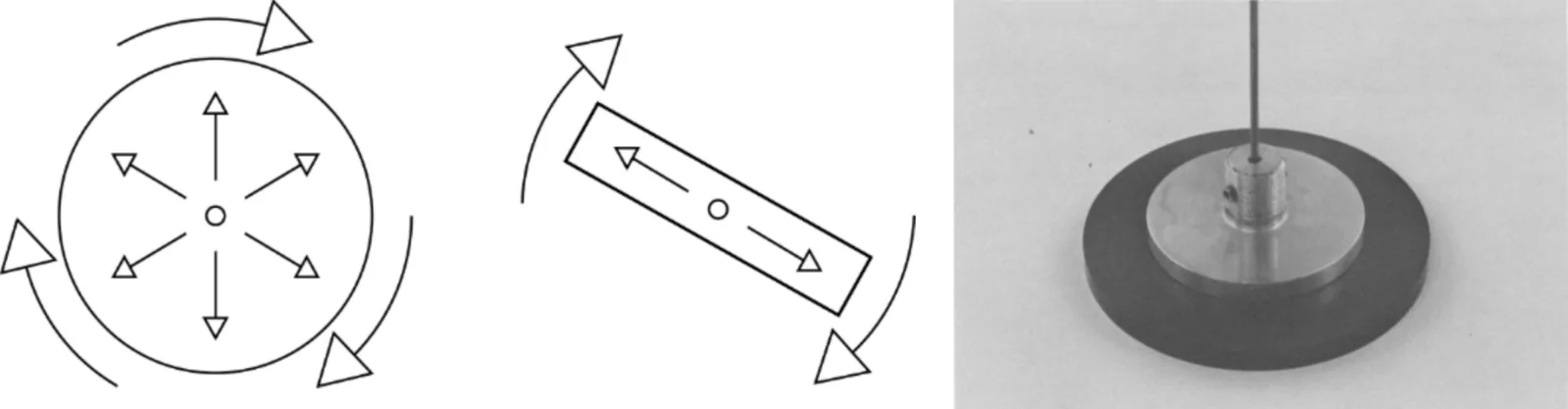
Spin testing, left: Schematic illustration of risk and bar samples, right: silicon carbide spin testing disk, bonded to an aluminium holding fixture with a flexible shaft (reprinted from [100] with permission of Springer Nature)

Spin testing, also referred to as a rotational fast fracture test, is widely used for stress testing of ceramic turbine rotors [98, 99], but has also been adapted for general strength testing of ceramics [100, 101]. In this test, a disk or bar shaped sample is spun rapidly, generating centrifugal forces which in turn result in tensile stresses throughout the sample [100] connected the samples to a 150.000 RPM air turbine using a flexible shaft, attached to a metal plate glued to the sample (Fig. 20). This allows the sample to rotate around its true center of mass without complicated alignment, thus preventing unwanted vibrations. The test was subsequently performed in a vacuum chamber to minimize air-resistance, while the sample was surrounded by a paraffin ring to catch fragments of the sample after fracture.

An analytical solution for the stress state in spun bar and disk samples was formulated by [100]. The stress distribution for the disk-shaped specimen is relatively complex, varying from fully biaxial at the center to mostly uniaxial at the edges, while for a bar-shaped sample the stress is uniaxial tension with increasing intensity towards the center. Results from the spin testing were compared with three-point bending tests by [100]. Compensating for sample size and stress distribution, a good correlation was found for silicon carbide samples. For alumina samples the results showed poor correlation; the authors argued that this might have been caused by fabrication differences between the samples. In [101], four-point and ball-on-ring bending tests were used to predict the spin test strength of Si_3_N_4_ ceramics using Weibull statistics, finding a satisfactory correlation.

## Discussion

A qualitative comparison is made between the selected test methods, based on the testing considerations described in Sect. "Testing considerations". The main advantages and limitations of the different tests are summarized in Table 2.

**Table 2:** Summary of advantages and limitations for the discussed methods

Test method	Advantages	Limitations
Uniaxial tensile test	- Widely applied, standardized - Uniaxial tension in bulk and surface - Sources of measurement error well-documented	- Complex grips required (risk of contact failure) - Highly sensitive to eccentricities - Relatively complex specimen geometry
Three-point bending	- Widely used, standardized - Sources of measurement error well documented - Simple specimen geometry - Self-aligning	- Maximum tension at surface - Small area subjected to maximum tension;
Four-point bending	- Widely used, standardized - Sources of measurement error well documented - Simple specimen geometry - Larger surface area subjected to uniform tension	- Maximum tension at surface
Ball-on-ring	- Symmetrical biaxial stress state - Simple specimen geometry - Limited risk of edge failure	- Maximum tension at surface - Limited area subjected to maximum tension - Stress intensity under loading ball load-dependent (Hertzian cracking) - Sensitive to geometric inaccuracies (uneven contact between ring and glass surface)
Ring-on-ring	- Symmetrical biaxial stress state - Simple specimen geometry - Widely used, standardized - Limited risk of edge failure - Sources of measurement error well-documented	- Maximum tension at surface - Sensitive to geometric inaccuracies (uneven contact between ring and glass surface) - Risk of invalid failure under the loading ring
Piston-on-three-ball	- Biaxial stress state - Simple specimen geometry - Limited risk of edge failure - Self-aligning	- Maximum tension at surface - Limited area subjected to maximum tension - Complex stress distribution - Precise stress state under piston unreliable - Precise alignment of sample parallel to loading piston required
Ball-on-three-balls	- Biaxial stress state - Simple specimen geometry - Limited risk of edge failure - Self-aligning	- Maximum tension at surface - Limited area subjected to maximum tension - Complex stress distribution - Precise stress state under piston unreliable - Stress intensity under loading ball load-dependent (Hertzian cracking)
Pressure-on-ring	- Biaxial stress state - Simple specimen geometry - Limited risk of edge failure - Minimal risk of failure at load application	- Maximum tension at surface - Sensitive to geometric inaccuracies (uneven contact between ring and glass surface) - Specialized non-standard equipment required
Brazilian disk test	- Simple testing equipment - Simple specimen geometry - Uniaxial tension in bulk and at surface	- Risk of unintended contact failure at the supports (risk can be reduced by using flattened disk or concave loading plates) - Explosive failure complicates post-fracture analysis - Strong compression stresses perpendicular to the tensile stress
Theta specimen	- Uniaxial tensile test without need for complex grips - Simple testing equipment	- Complex specimen geometry - Reliability of fracture in gauge section uncertain
Diametral sphere compression	- Biaxial tension fully in bulk - Simple testing equipment - Simple specimen shape, minimal post-processing of surface required	- Failure mechanism uncertain - Explosive failure complicates post-fracture analysis
Spin testing	- Can apply uniaxial and biaxial stress states - No complex gripping mechanism required - Minimal risk of contact failure on loading	- Specialized equipment required - Explosive failure complicates post-fracture analysis
Ultrasonic testing	- No complex gripping mechanism required - Minimal risk of contact failure on loading - Can accommodate geometric deviations - Simple rod or bar specimen shape for glass - Scalable	- Complex specialized equipment required - Extensive data post-processing required - Novel, with limited use

### Stress Distribution, Stressed Volume and Scalability

The various tests have been clustered based on the stress distribution they impose on a sample, i.e. whether the maximum tensile stresses occur at the material surface, bulk, or both. For instance, the various flexural tests generate a stress gradient where the largest stresses occur at the material surface, while the tests that apply a uniform stress apply a maximum stress at both the surface and bulk of the specimen. Uniquely, the sphere compression test applies a maximum tension fully in the material bulk, theoretically without stressing the material surface [89]. An overview of the various setups and their stress distributions is given in Table 3. An additional sub-cluster is based on the amount of material subjected to maximum tension in each setup, relative to the size of the specimen.

**Table 3:** Comparison of tests based on location of maximum stress, and the stressed area or volume relative to the size of the specimen

Surface, small area	Surface, large area	Surface and bulk, small volume	Surface and bulk, large volume	Bulk, small volume
Three-point bending	Four-point bending	Brazilian disk	Uniaxial tensile test	Sphere compression
Ball-on-ring	Ring-on-ring	Theta specimen	Ultrasonic tensile test	
Ball-on-3 balls	Pressure-on-ring		Spin test	

The ease with which the amount of material tested can be scaled up or down varies between setups. Due to their relative simplicity, three- and four-point bending setups have been applied across a wide range of scales, from small material samples [53] to large scale beams [102], covering several orders of magnitude. The uniaxial tensile can be used to test various volumes of material bulk by using test samples of various lengths, though significantly changing the cross-section of the tensile sample will make the specimen incompatible with standardized gripping systems. In contrast, the loading mechanism of the ultrasonic tensile test should theoretically allow for the testing of specimen at widely different scales, as long as a suitable natural frequency can be achieved with the samples [96, 97]. However, such scaling has thus far not been attempted in practice. The biaxial flexural tests can be scaled to accommodate different loaded surface areas, though the specimen thickness should be chosen so that samples align with simple plate theory [54]. The Brazilian disk, sphere compression test and Theta specimen have been used at scales ranging from (sub-)millimeter [74, 84, 90] to several centimeters [82, 86, 94]. At a larger scale, the disk and sphere specimen can be expected to show even stronger explosive failure due to the high force required to initiate fracture, while the larger thetas become challenging to fabricate. For the spin test, the obtained stress is based on the specimen length in the case of a beam, and the diameter in the case of a disk geometry, in combination with the rotation speed [100]. The size of specimens that can be tested is therefore dependent on the rotation speed that can be reached in combination with the size of the vacuum chamber available.

When characterizing the bulk strength of glass in the presence of bulk flaws, ideally a test should be used that applies a maximum tensile stress in the bulk, thereby minimizing the chance of unintended failure from the material surface. Due to this, the sphere compression test is interesting as it applies a tensile stress that is located fully within the bulk of the material, while minimizing surface stresses. This behavior was used by [94] to investigate a bulk-located flaw under tensile loading. An alternative approach of increasing the probability of surface failure is the ultrasonic tensile test, which has a very short time to failure (4–7 ms for graphite) (Pagnoncelli, Paolino, et al., 2024). Consequently, environmentally assisted slow crack growth is negligible and surface flaws approach their inert strength, while bulk flaws are less affected, thereby further increasing the likelihood of bulk failure.

Methods that apply a tensile stress to both the surface and bulk can be used to test bulk flaws, as long as the finish of the surface is of sufficient high quality relative to the severity of the bulk flaws, thereby avoiding surface failure. Failure from bulk flaws are regularly observed in flexural tests on advanced ceramics [25], despite the maximum stresses being located at the surface rather than in the bulk. In the case of cast glass however, even with multi-step surface polishing up to 600 grit, failure in four point flexural testing has been found to occur from the material surface or edges, despite the presence of notable bulk flaws [4, 16]. Further refinement of the specimen surface should lead to more reliable failure from bulk flaws, however this level of sample preparation can be impractical when a large number of specimens is required.

### Stress State

The different tests in this review are designed to impose a uniaxial or biaxial tensile stress state; as shown in Table 4. The Brazilian disk and sphere compression tests apply a significant compressive stress in addition to the uni- or biaxial tensile stresses [76], complicating the behavior of the material and flaws. The spin test can be used to apply either a uniaxial or biaxial tensile stress, by using a bar or disk sample, respectively [100]. For the fundamental research into bulk flaws in glass, it is valuable to explore both stress states.

**Table 4:** Comparison of tests based on applied stress state

Uniaxial tension	Biaxial tension
Uniaxial tensile test	Ring-on-ring
Three-point bending	Ball-on-ring
Four-point bending	Ball-on-3 balls
Brazilian disk	Pressure-on-ring
Theta specimen	Sphere compression
Ultrasonic tensile test	Spin test
Spin test	

### Test Outputs

The different data obtained from the various methods are summarized in Table 5. The majority of tests provide force–displacement data, which in the case of brittle solids such as glass will consist of a linear elastic region followed by a sudden drop at fracture. From this data strength (and in some cases Young’s modulus) can be derived. Notable exceptions are the ultrasonic tensile test and the spin test. During UST testing, the strain at several points in the sample is measured. A numerical optimization of the material properties is then performed in a FEA model to match the observed strains. Through additional optimization steps, other properties such as material density and Poisson’s ratio can also be determined [96, 97]. For the spin test, the revolutions per minute at failure is recorded, from which the failure stress can be derived analytically [100] or numerically [99].

**Table 5:** Data outputs and resulting properties

Test setup	Data output	Material properties measured
Uniaxial tensile test	Force, displacement, time	Uniaxial tensile strength, E modulus
Three-point bending	Force, displacement, time	Uniaxial flexural strength, E modulus
Four-point bending	Force, displacement, time	Uniaxial flexural strength, E modulus
Ring-on-ring	Force, displacement, time	Biaxial flexural strength
Ball-on-ring	Force, displacement, time	Biaxial flexural strength
Ball-on-three balls	Force, displacement, time	Biaxial flexural strength
Pressure-on-ring	Pressure, time	Biaxial flexural strength
Brazilian disk	Force, displacement, time	Uniaxial tensile strength, E modulus^1^
Theta specimen	Force, displacement, time	Uniaxial tensile strength
Sphere compression	Force, displacement, time	Biaxial tensile strength, compressive capacity^2^
Ultrasonic tensile test	Specimen strain (amplitude and ramp), time	Uniaxial tensile strength^3^, E modulus, density^3^, Poisson's ratio^3^
Spin test	RPM, time	Uni/biaxial strength, Allowable RPM^2^

^1^In combination with strain gauges or DIC

^2^Component rather than material property

^3^Can be obtained through numerical material property optimization

### Accuracy, Consistency and Verifiability

Different tests are compared on the expected accuracy and consistency of their measurements, as well as the possibility of verifying results through post-fracture analysis. An overview is given in Table 6.

**Table 6:** Comparison of tests based on accuracy, consistency and verifiability

Standardized, compatible with fractography	Behavior well-understood, compatible with fractography	Some uncertainties, fractography challenging	Strong uncertainties, fractography challenging
Uniaxial tensile test	Pressure-on-ring	Theta specimen	Sphere compression
Three-point bending	Ball-on-ring	Brazilian disk	
Four-point bending	Ball-on-3 balls	Ultrasonic tensile test	
Ring-on-ring		Spin test	

Some of these tests have been extensively used for brittle solids. This category includes the uniaxial tensile test [36], three- and four-point bending tests [45, 46, 49], and the ring-on-ring test [62, 65]. As a result of this, sources or measurement bias these have been widely reported, and measures to prevent this are known [25, 42, 54]. The failure behavior in these tests can be verified using fractography, as the samples fail at a relatively low strain energy, which limits damage to the sample at fracture, resulting in predictable fracture patterns [22].

The pressure-on-ring test has a stress state and distribution that is similar to the ring-on-ring setup, while having the added advantage of having a reduced risk of unintended failure at the loading contact points, as the load is introduced over a large area [58, 61]. However, due to the specialized setup required, this test has seen limited use in practice, and consequently no standardized test guidelines are available.

The ball-on-ring and ball-on-three-balls setups rely on Hertzian fracture beneath the loading ball to establish contact with the sample, with the precise behavior being dependent on the diameter of the loading ball and the mechanical properties of the tested material. No complete analytical solution for the stress state under the loading ball exists, requiring FEA modelling to obtain an accurate stress state [71].

The spin test is widely used for the functional testing of components [98, 99], but has seen limited use as a material testing technique [100, 101]. As the load applied directly to the specimen is relatively low, there is a low risk of unintentional contact failure, however, care is needed to ensure the specimen remains well-balanced during spinning. Spin test samples burst explosively and can sustain secondary damage due to collisions with the test chamber, which make it challenging to perform fractography. This can be alleviated to some extend through additional measures, such as surrounding the samples in paraffin or gelatin [100].

For the Brazilian test, a risk for failure from Hertzian fracture at the loading points can potentially cause invalid results. Different measures to mitigate this risk include flattened contact surfaces, curved loading jigs or soft interlayers. The test has been standardized for materials with relatively low tensile strengths, fracture toughness and/or stiffness (e.g. natural rock [75], concrete [73] and graphite [74]), generally splitting in several larger fragments, where it is relatively easy to determine whether a valid fracture in the center of the specimen occurred. For glass, fracture is much more explosive [79] which makes it challenging to verify the test results. High-speed recordings of soda-lime glass disks under (semi-)static loading indicates valid fracture occurring in the center of the sample [77].

The sphere compression test, is potentially unreliable because is no agreement in literature about the governing failure mechanism (discussed in Sect. "Diametric sphere compression"), and fractography cannot be performed to verify this due to the explosive nature of failure it induces. Additional measures to retain fragments in, for example, paraffin or gelatine are possible [88], but complicate the testing process; while post-fracture analysis will remain difficult due to the large number of fragments [88, 94]. Even if fracture through tension in the bulk can be confirmed, the critical flaw where fracture originates is difficult to identify. New developments in digital image processing and computer vision might offer new possibilities for reconstructing these samples, but need further development. Research by Wang et al. [94] has shown that a sufficiently severe controlled flaw in the material bulk of a glass sphere reduces its strength significantly, and observed crack growth in the bulk leading to failure. Failure was also less explosive, with the sphere fracturing into several large fragments. However, this behavior is expected to be inconsistent with the presence of more varied and irregular flaws that are present in for example recycled glass. Some of the measures used in the Brazilian disk test to reduce the probability of contact fracture could potentially be applied to a sphere compression test; for example, using a specimen with two flattened loading surfaces, or using a concave loading plate. To the knowledge of the authors, such modified sphere compression tests have yet to be attempted.

**Fig. 21:**
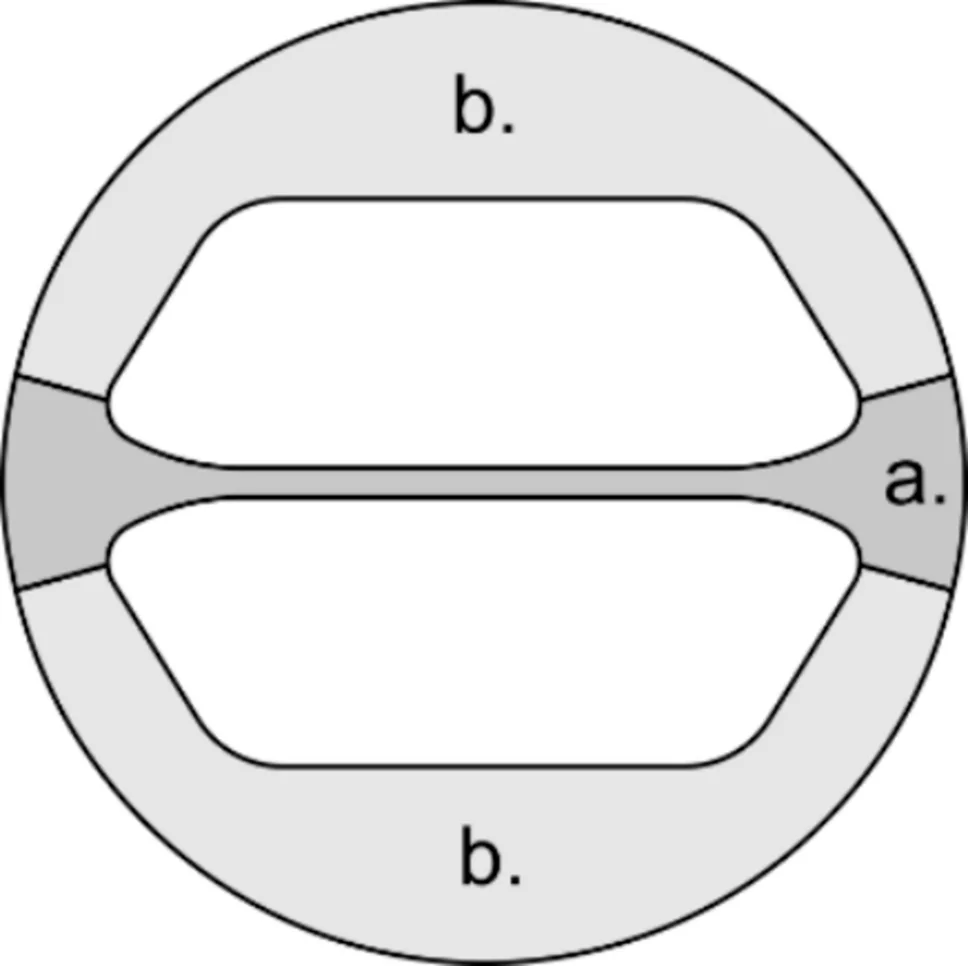
Proposal of a ‘hybrid’ theta samples, fusing contaminated glass in the gauge section (**a**) with higher purity glass in the remainder of the sample (**b**), to reduce the risk of invalid failure, occurring outside the intended gauge section

For the theta specimen, fracture has to first occur in the central gauge section to provide a valid test result. However, these specimen always fracture at several points [84, 86], which can make it challenging to ascertain that a valid fracture has occurred. This is expected to become even more of a challenge when a material with significant flaws is tested (such as recast waste glass), as there is a notable risk of a critical flaw being located outside the gauge section, causing invalid fracture. A hybrid sample can be imagined that alleviates this, where the glass composition of interest is confined to the middle strip of the Theta, while the rest of the Theta sample is made of higher quality, uncontaminated glass (Fig. 21). This however further complicates the manufacturing process, and introduces new risks, such as imperfect fusing between the two glass types. Further investigation is required for such a hybrid setup.

For the theta specimen, different methods can be used to ascertain whether valid fracture in the gauge section of theta specimen test, e.g. high-speed camera recordings (potentially in combination with DIC), interruption of conductive paint [83] or fracture mirror measurements [86], however these are relatively complex solutions that complicate the testing process.

The ultrasonic tensile test has not been explored extensively, but appears to provide results in line with other test methods. At fracture, extensive fracturing occurs around the failure location, which makes it challenging to perform post-failure analysis [96, 97] combined UST testing with micro-CT scans of the samples before and after failure, which allowed them to identify the fracture origin.

### Specimen Complexity

This concerns the practicality of fabricating the required test specimen based on the complexity of the geometry. Ease of fabrication is largely dependent on the material and the method it is fabricated i.e. disk geometries cut from materials that are produced in the form of rods or drilled core samples. This comparison is made assuming cast glass, based on the possibilities and limitations outlined in Section "Background". Through glass casting, complex three-dimensional geometries can be manufactured that might be challenging to achieve in other brittle materials such as pressed ceramics. However, unless a high-precision, non-reactive mold is used, polishing is required to achieve a high-quality surface finish [4]. This is difficult to reliably achieve for geometries with reentrant corners or holes (as shown for example with the glass theta specimens explored in [86]). Complex geometries can be used in glass, as long as these are fabricated in a way that requires limited or no post-processing, e.g. casting them in high-precision, non-reactive molds.

For this comparison, a simple geometry is defined as having surfaces that are flat or have a constant curvature. This includes geometries such as flat plates, rectangular beams, rods, disks and spheres. Complex geometries are defined by having facets such as setbacks, undercuts, reentrant corners or holes, including geometries such as dogbones or the theta specimen. An overview is given in Table 7.

**Table 7:** Comparison of tests based on geometrical complexity of specimen required

Simple	Complex
Three-point bending	Uniaxial tensile test
four-point bending	Theta specimen
Ring-on-ring	Ultrasonic tensile test
Ball-on-ring	
Ball-on-3 balls	
Pressure-on-ring	
Brazilian disk	
Sphere compression	
Ultrasonic tensile test	

The ultrasonic tensile test is placed in both categories, as the specimen shape is dependent on the stiffness, mass and tensile strength of the tested material to reach the natural frequency and tensile stresses required for a successful test. For some materials (such as glass and alumina) this can be achieved with a simple rod or beam geometry [97], while for other materials (such as graphite) a more complex geometry such as a dogbone is necessary [96].

### Dimensional Accuracy

Dimensional accuracy concerns the sensitivity of the tests to geometrical deviations of the samples. An overview is given in Table 8. The tests in this section are considered from low to high sensitivity.

**Table 8:** Comparison of tests based on dimensional accuracy of samples required

Low	Medium	High
Three-point bending	Four-point bending	Uniaxial tensile test
Ball-on-3 balls	Ring-on-ring	
Pressure-on-ring	Theta specimen	
Ball-on-ring	Spin test	
Brazilian disk		
Sphere compression		
Ultrasonic tensile test		

Some test setups are designed to consistently apply loads to the sample despite geometrical deviations as they require only limited contact with the test machine, i.e. are self-aligning. This includes the three-point bending tests, the four-point bending test with articulating loading fixture, ball-on-three-balls test, Brazilian disk and sphere compression tests. For the ultrasonic test, the sample is stressed by letting it resonate at its own natural frequency, instead of being subjected to an external tensile load, which limits alignment issues due to geometric deviations [96]. The biaxial flexure tests that use support rings (ball-on-ring, ring-on-ring and pressure-on-ring) are more sensitive to geometric deviations, as incomplete contact between the support ring and the specimen can occur during loading, though this effect can be partly compensated for by using a soft interlayer between the rings and the sample [54]. The pressure-on-ring has a lower risk of contact failure at the loading points compared to the ring-on-ring setup, as the flexible loading membrane can accommodate geometric deviations [58, 61].

The theta specimen can accommodate some geometric deviations. However, there is a risk of unintended stress gradients developing around the central gauge section, either due to uneven contact at the loading contact points, or due to shape deviations around the gauge section [86].

Geometric deviations In the spin test could potentially lead to the sample destabilizing during testing. The setup proposed by [100] has some self-balancing capabilities by suspending the sample from a flexible quill, which limits the sensitivity to geometric deviations. The precise level of deviation that can be tolerated this way has not been explored in great detail.

The uniaxial tensile test is highly sensitive to geometric deviations, as discussed in Sect. 0. Small eccentricities can lead to significant bending stresses deviating from a uniform tensile stress state [42], while geometric imprecisions in the gripped sections can lead to uneven loading and local contact failure. Bending stresses due to misalignment can be limited through articulated grips. Friction in the articulated grips should be minimized, particularly for relatively low-strength materials [42], which includes glass.

### Availability of Tools and Equipment

A practical consideration that can steer the choice of test setup is the availability of the equipment needed. For this overview, three categories are distinguished: setups that have been standardized and are can be considered widely available (generally using universal testing machines (UTM) with common attachments), setups that require some specialized attachments (e.g. specialized grips for tensile testing), and finally setups that require custom, non-standardized tools and equipment. An overview is given in Table 9.

**Table 9:** Comparison of tests based on availability of equipment required

Common	Uncommon	Special
Three-point bending	Uniaxial tensile test	Pressure-on-ring
Four-point bending	Ball-on-ring	Ultrasonic tensile test
Ring-on-ring	Ball-on-3 balls	Spin test
Brazilian disk		
Theta specimen		
Sphere compression		

## Conclusion

This review explored various tensile testing methods for brittle materials to identify test that are feasible for testing the bulk strength of glass in the presence of bulk flaws. Based on this, three tests are recommended.

For uniaxial loading, the uniaxial tensile test is recommended. This is the most direct test to apply a uniform tensile stress. It displays a simple fracture pattern that is compatible with fractographic verification. Previous work by [34] has shown the feasibility of this setup for testing volumetric glass, while the standardized guidelines for advanced ceramics in the ASTM C 1273 [36] can be used as a guideline for further developing a testing methodology for glass. The principal challenge is the fabrication of the test specimen due to the high level of fabrication accuracy required, in combination with the more complex geometry required for gripping, which complicates post-processing. For such a result, it is recommended to cast the specimens in a high-quality, non-reactive mold. To minimize eccentric loading effects, a low-friction articulating gripping setup is recommended. The presence of unintended stress gradients can be identified through DIC, or polarized light strain visualization if specimen is sufficiently transparent.

A potential alternative for uniaxial testing is the use of the ultrasonic fatigue machine, commonly used for rapid fatigue tests, to perform ultrasonic tensile tests (UST). It avoids the strict requirements for the test specimens encountered in the uniaxial tensile test, as it can accommodate small geometric inaccuracies in the specimen, and can use a simple geometry for glass. The short time to failure minimizes the influence of environmentally assisted slow crack growth, making the specimen less susceptible to surface failure, and more likely to fracture from the bulk. Preliminary tests have shown that this setup is compatible with glass. However, as a relatively novel test method, there are still uncertainties that warrant further testing. Due to the short time to failure, this is essentially a semi-static test; it should be investigated whether the measured bulk properties are representative of results obtained with quasi-static tests. Furthermore, the extensive damage that occurs around the fracture zone complicates fractographic analysis. Finally, at present, the UST requires a specialized and complex test setup and extensive data post-processing. Therefore, despite its strong potential, the above-discussed limitations may restrict the broader use of this setup.

For biaxial loading of bulk flaws, both the spin and the sphere compression test are suitable for applying a uniform equibiaxial stress state in the material bulk. Comparing these, the spin test stresses both the material surface and bulk, while the sphere test uniquely applies a tensile stress fully in the bulk of the material, thus more likely obtaining failure from the bulk. Additionally, the sphere compression test can be performed using a standard UTM, while the spin test requires specialized equipment. Finally, the sphere geometry is compatible with glass fabrication. Therefore, the sphere compression test merits further investigation. The notable shortcoming of this test are the uncertainties about its failure mechanism. It was shown in [94] that this method can induce bulk failure in the presence of a severe bulk flaw. However, the failure behavior in less severe and more randomly distributed requires further investigation. Some measures that reduce the risk of contact failure might be taken from the Brazilian disk tests, such as flattened contact surfaces, or concave loading plates.

## Data Availability

No datasets were generated during the current study. Any analysed data is available in the referenced studies.

## Footnotes

1 Volumetric glass is defined here as glass of substantial thickness and three-dimensional form, in contrast to flat or thin-walled elements such as float glass and containers.

2 Flaws in the bulk resulting from foreign matter (e.g. of different glass composition or metal contamination) can be in the form of either crystalline inclusions or glass inhomogeneities.

3 However, ASTM notes that the preferred method for testing the tensile strength of graphite it through a direct tensile test following ASTM C749, and the disc compression method should only be used if a tensile sample cannot be fabricated due to material constraints.
